# Biomaterials in Tendon and Skeletal Muscle Tissue Engineering: Current Trends and Challenges

**DOI:** 10.3390/ma11071116

**Published:** 2018-06-29

**Authors:** Megane Beldjilali-Labro, Alejandro Garcia Garcia, Firas Farhat, Fahmi Bedoui, Jean-François Grosset, Murielle Dufresne, Cécile Legallais

**Affiliations:** 1 CNRS, UMR 7338, Biomécanique-Bioingénierie, Sorbonne Universités, Université de Technologie de Compiègne, 60200 Compiègne, France; megane.beldjilali-labro@utc.fr (M.B.-L.); alejandro.garcia-garcia@utc.fr (A.G.G.); firas.farhat@utc.fr (F.F.); jean-francois.grosset@utc.fr (J.-F.G.); murielle.dufresne@utc.fr (M.D.); 2 CNRS FRE 2012, Laboratoire Roberval, Sorbonne Universités, Université de Technologie de Compiègne, 60200 Compiègne, France; fahmi.bedoui@utc.fr

**Keywords:** collagen, sponge, electrospinning, stem cells, elastic modulus, stretching

## Abstract

Tissue engineering is a promising approach to repair tendon and muscle when natural healing fails. Biohybrid constructs obtained after cells’ seeding and culture in dedicated scaffolds have indeed been considered as relevant tools for mimicking native tissue, leading to a better integration in vivo. They can also be employed to perform advanced in vitro studies to model the cell differentiation or regeneration processes. In this review, we report and analyze the different solutions proposed in literature, for the reconstruction of tendon, muscle, and the myotendinous junction. They classically rely on the three pillars of tissue engineering, i.e., cells, biomaterials and environment (both chemical and physical stimuli). We have chosen to present biomimetic or bioinspired strategies based on understanding of the native tissue structure/functions/properties of the tissue of interest. For each tissue, we sorted the relevant publications according to an increasing degree of complexity in the materials’ shape or manufacture. We present their biological and mechanical performances, observed in vitro and in vivo when available. Although there is no consensus for a gold standard technique to reconstruct these musculo-skeletal tissues, the reader can find different ways to progress in the field and to understand the recent history in the choice of materials, from collagen to polymer-based matrices.

## 1. Introduction

The most advanced studies on tissue engineering (TE) concerning the musculo-skeletal system focus on bone and cartilage tissue engineering [1,2,3]. TE aims at better understanding and mimicking the intrinsic properties of each tissue and its interface, such as the complete regeneration of the enthesis [4]. Applications on tendon and muscle tissues are less widespread and still emergent, with various approaches that are still far from clinical applications, but very useful for progress in understanding these specific tissues. The numerous parameters that influence the biological or mechanical outcomes make it uneasy to derive any experimental rationale. This lack of rationale has hampered the emergence of a gold standard experimental protocol for the reconstruction of such biohybrid tissues.

**Figure 1:**
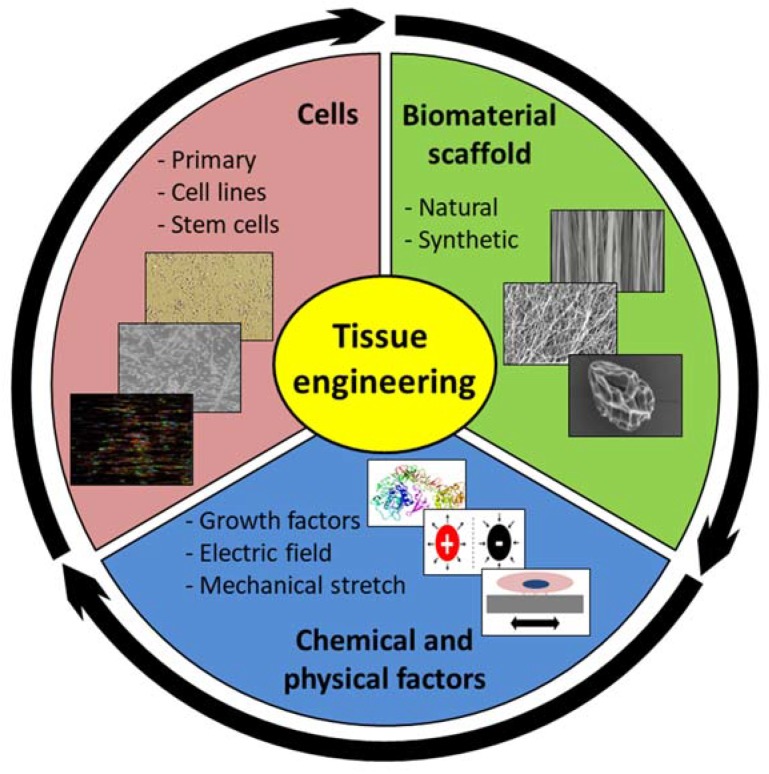
The three pillars of tendon/muscle tissue engineering: cells are cultured on a scaffold where they can attach, proliferate, or differentiate, giving them a phenotype relevant for the renewal of tissue functions. The mechanical and biochemical environments are of prime importance for triggering specific responses.

Therefore, to unite the efforts that are made by the various teams, the present review focuses on tissue engineered reconstructions of tendon and skeletal muscle tissues, as well as the myotendinous junction (MTJ), which is a key element for further implantation. As with other forms of tissue engineering, muscle or tendon tissue engineering relies on three pillars: cells, biomaterials, and environment, ensured by chemical or physical factors (Figure 1). Bioreactors are often required to perform three-dimensional (3D) cultures and mimic the cells’ in vivo niche and environment, while ensuring the better control of cell culture conditions and possibly inducing cell responses to mechanical stimuli.

**Figure 2:**
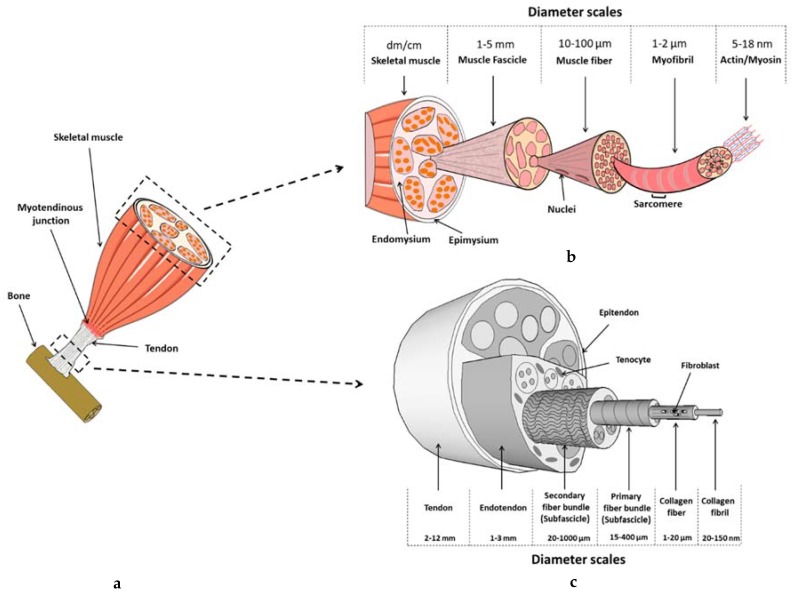
Overview of the bone-tendon-muscle continuum in the human musculo-skeletal system (**a**). Multi-scale description of a skeletal muscle (**b**) and a tendon (**c**).

For this review, we have chosen to present biomimetic or bioinspired strategies that are based on an understanding of the native tissue structure/functions/properties of the tissue of interest. We postulate that in-depth understanding of the native functions of muscle and tendon, as well as their alterations, should guide the research program leading to their reconstruction. These two tissues are involved in the transmission of efforts to bone tissue, ensuring body motion. Interestingly, they have the same embryogenic origin and present similarities in their multi-scale organization, but also have differences at various levels (Figure 2), which will lead to completely different approaches in terms of tissue reconstruction. Therefore, to highlight the efforts that are made to understand native structures, the first part will present the multi-scale organization of the tissue of interest (tendon or muscle), followed by a second part showing the alterations, leading to the need for reconstruction. Then, we will provide information about the various types of materials, cells, and environment (the three pillars) that have been assessed for bioconstruction, and propose a classification. Finally, we will show how the shape of the materials themselves, which is made possible by means of different production techniques, can guide not only the structure and mechanical properties of the scaffold, but also the biological responses, and we will analyze to what extent these integrated approaches can lead to a functional reconstructed tissue.

## 2. Tendon

### 2.1. Tendon Composition and Structure

Tendons are specialized fibrous tissues that join skeletal muscle to bone and make body motion possible through the forces that are generated by the skeletal muscles to bone tissues [5]. They act as highly adapted elastic springs that stretch and store energy, which returns to the system through elastic recoil, thus improving locomotor efficiency. This function is closely related to the tendon’s composition and structure. Tendon is a dense, connective tissue with limited cell content, vascularization, and innervation [6]. The main component of tendon is water (60% to 80% in weight) [7].

Collagen represents the major component (60% to 85% dry weight) of the extracellular matrix (ECM), type I collagen being the most abundant and responsible for the fibrous structure [8]. Type I collagen molecules aggregate to form collagen fibrils, the basic nanostructural tendon unit. Bundles of fibrils form fibers, fibers group into fiber bundles or fascicles; and, fascicles bundle together within connective tissue sheaths (endotenon) to form larger bundles that are surrounded by another connective tissue sheath (epitenon) [9] (see Figure 2). Collagen fibers display a wave pattern, which is also known as a crimp [10]. Non-fibrous molecules are present on each level, the main ones being proteoglycans (PGs) [11,12,13,14,15], such as decorin [11,16] and aggrecan [17]. ECM also contains glycoproteins, including tenascin-C and fibronectin [18,19].

Tendon cells are key players in tendon growth, maintenance, adaptation to changes in homeostasis, and remodeling in the case of minor or more severe disturbances to tissue. The cells are responsible for the synthesis and turnover of tendon ECM components and its related structure. Mature tendon contains predominantly tenocytes/tenoblasts [20], which account for around 90–95% of the cell population. Tenocytes are terminally differentiated cells typically anchored to the collagen and located throughout tendon tissue. Tenoblasts are immature tendon cells that give rise to tenocytes. Recently, a new cell type has been characterized in tendon tissue: resident tendon stem/progenitor cells (TSPC). TSPCs represent 1–4% of tendon resident cells, and they exhibit the same characteristics as adult mesenchymal stem cells (MSC) [21].

**Figure 3:**
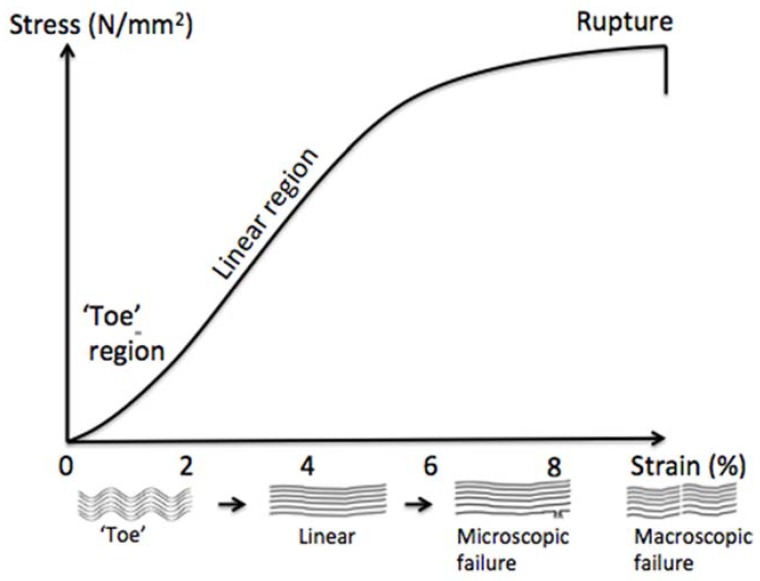
Typical tendon response to stretching at fixed strain rate: stress-strain curve illustrating the various deformations of the collagen fibrils.

Regarding the composition and structure of the ECM, tendon appears to be an anisotropic and viscoelastic material that is capable of resisting high tensile forces [22]. At a fixed strain rate, the stress-strain curve of tendons has three distinct regions (Figure 3). The tendons stress strain response is strain rate dependent leading to higher stiffness and lower strain break while keeping the same chronological damage process when stretched at high strain rate [23]. The toe region corresponds to low strains (<2%), where the crimp structure is straightened. Once the collagen fibrils have been straightened, the load-deformation relationship becomes relatively linear, representing the physical stretching of the collagen fibrils (~2–4%). Beyond this region, additional loading causes micro failures to individual fibrils up to a failure of the whole tendon over ~8% of strain [24]. The in vivo evaluation of human tendon mechanical properties depends on the investigation method (ultrasound, magnetic resonance imaging) and stretching protocols used. For human tibialis anterior and gastrocnemius tendons, Maganaris et al. (2002) [25] calculated an elastic modulus (EM) around 1.2 GPa, while an EM value of 600 MPa was reported for the patella tendon [26].

### 2.2. Tendon Injuries and Healing

As a result of physical activity (sport or professional activities), trauma, or aging, tendinopathies, which is a clinical syndrome characterized by the combination of pain, swelling, and impaired performance, are an increasing health problem that affects an estimated number of 100 million people worldwide annually [16]. Owing to its hypovascularity and hypocellularity, tendon has a weak intrinsic healing ability and it often responds poorly to pharmaceutical treatments [20]. Thus, total repair requires prolonged rehabilitation in most cases. Tendon healing follows three well-described steps: inflammatory, proliferative, and remodeling phases, the latter characterized by the alignment of collagen fibers parallel to the muscle force direction, which determines the recovery of the tendon tissue’s biomechanical properties [27]. The biomechanical cues for repaired tissue are mostly inferior to those of native tissue, causing an increasing rate of tendon re-injuries. To overcome the inability of the repaired tissue to regenerate the functions of native tendon, and to improve healing rates, surgical approaches, such as sutures or transplantation of autografts, allografts, or xenografts have been described and clinically performed. Autografts remain the gold standard for surgical procedures for tendon repair. Alternatives, such as: (1) allografts such as GraftJacket™ (Wright Medical Technology, Arlington, TN, USA) or AlloPatch HD^®^ (MTF Sports Medicine, Edison, NJ, USA); (2) xenografts, such as TissueMend^®^ (Stryker Howmedica Osteonics, Kalamazoo, MI, USA) or CuffPatch^®^ (Arthrotek, Warsaw, IN, USA); and, (3) artificial prostheses, such as STR Graft™ (Biorez Inc., New Haven, CT, USA) or SeriCuff™ (Serica Technologies, Medford, MA, USA) have been developed and commercialized [28]. However, these approaches usually result in fibrotic tissue with low mechanical properties when compared to native tendon, and so far none of these techniques has provided complete healing for tendon disorders [29].

### 2.3. Tendon Tissue Engineering

Tissue engineering is a promising alternative to the natural healing process for tendon repair, especially in the reconstruction of large damaged tissues. The inability of native tendon to neosynthesize ECM is expected to be overcome by the design and production of a scaffold that hosts cells differentiated into a tendon lineage.

**Figure 4:**
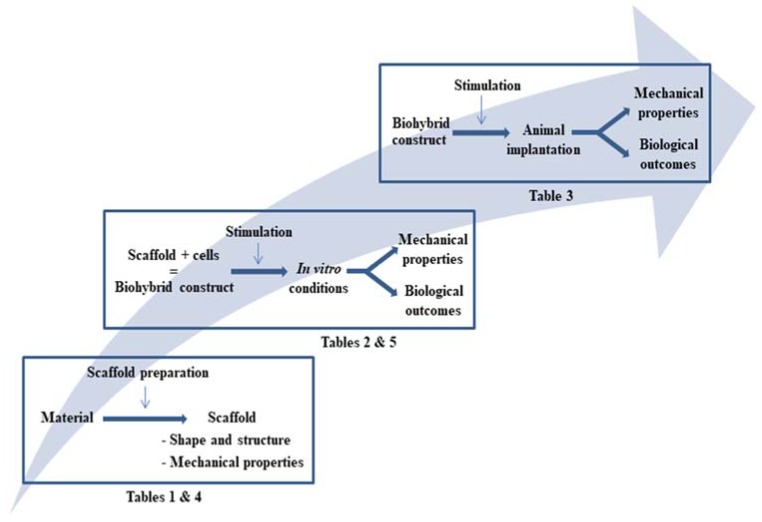
Rationale for the choice of studies and contents reported in the tables, for tendon, and muscle tissue engineering, respectively.

After reviewing the literature on the approaches that were adopted in this field in the last fifteen years, we present the papers selected in three tables (Figure 4).

Table 1 is dedicated to a summary of details of the major materials and methods, including, if present, the mechanical characteristics of the scaffold. Table 2 focuses on in vitro studies performed with the same scaffolds, identifying, if present, the effect of physical stimulation. Finally, Table 3 provides the in vivo outcomes, i.e., the behavior of the same TE constructs after their implantation into animal models, when available. After an analysis of the selected articles over the period of interest, we decided to only select those in which an in vitro/in vivo application was presented, and which were detailed enough to bring up trends for current progress in research in the field. The list was ordered according to the shape of the scaffolds. In the following chapters, we will first briefly focus on the three pillars of tendon TE (in Section 2.3), to outline the major trends and guidelines, and are provided in Section 2.4, the mechanical and biological outcomes arising from the tendon biohybrid reconstructed tissues. Current research mainly focuses on obtaining mechanical properties that are similar to those of native tendon, and on efficient cell differentiation into tenocyte lineage, capable of producing a new ECM.

#### 2.3.1. Cells

Several cell sources can be used for tendon tissue engineering (Table 2). Adult mesenchymal stem cells (MSCs) are a promising cell source as they present the potential for self-renewal, clonogenicity, and multi-lineage differentiation, including tenogenicity. They regulate the inflammation response through the secretion of paracrine factors, and exhibit an immunomodulatory effect, which avoids immunosuppressive treatments after allogenic transplantation. MSCs can be extracted from a variety of tissues, including bone marrow (BMSC), adipose tissue (ASC), or directly from tendon [21]. BMSCs are the most widely-used stem cells in tendon engineering [30,31,32,33,34,35,36,37,38,39]. Related to BMSCs, ASCs are present in great quantities in adipose tissues and are harvested by less invasive techniques [40]. Recent work has shown that ASCs have a minor tenogenic differentiation capacity when compared to BMSCs, in vitro and in vivo after implantation in nude mice [41]. To drive the tenogenic differentiation of BMSC and ASC, adding different growth factors and differentiation factors to the culture medium has been used with success [42].

A murine pluripotent cell line, C3H10T1/2 is another relevant stem cell model [43] used in embryology and tendon repair studies [44], also employed by several teams in tendon engineering approaches [45,46,47].

Tendon Stem/Progenitor Cells (TSPCs) are quite heterogeneous and present common features with adult MSCs. Even if their roles in tendon healing and maintenance remain unclear, these cells are a promising tool in tendon engineering [21,30,48]. Isolated from the mid-substance of patellar tendon, TSPCs may be characterized by various markers [48]. TSPCs have the advantage of having inherent pro-tenogenic abilities and being an autologous source of cells. When compared with BMSCs, TSPCs display the highest levels of tendon-related markers (scleraxis, tenomodulin, cartilage oligomeric matrix protein, and tenascin-C), high clonogenecity, and proliferation. When injected into the injured tendon region in a rat model, TSPCs pretreated in vitro with pro-tenogenic differentiation molecules improve tendon repair [49]. However, they have the same disadvantages as tenocytes, i.e., their scarcity in tendon tissue and a risk of morbidity at the site of tissue extraction [50].

Tenocytes are terminally differentiated tissue-resident cells, which are responsible for the synthesis and homeostasis of the components of the ECM of tendons. Despite the advantages of using autologous cells and the cell type in charge of intrinsic healing tendon [51,52,53,54,55], the use of tenocytes raises a series of obstacles: limited capacity to proliferate, scarcity of donor tendons from which tenocytes can be extracted, low quantity of tenocytes in tendons that make them difficult to collect, cell de-differentiation processes during culture expansion, and a risk of major donor site morbidity [56]. To overcome these limitations, dermal fibroblasts (DFs) have been proposed as an alternative source of cells for tendon reconstruction as it is relatively easy to extract and expand them, and, thanks to their high potential, produce ECM components from them [57]. However, using DFs can result in scar formation, leading to poor mechanical properties when compared to native tissue [58].

#### 2.3.2. Modulation of the Environment

##### Biochemical Stimulation

Once tendons suffer from an injury, a cascade of events takes place to repair the damaged tissue. Cytokines and growth factors that are released by tendon cells or inflammatory cells recruited into the damaged area play a key role during the early phase of tendon healing via the induction of cell proliferation, ECM synthesis, and remodeling [59]. Of these factors, vascular endothelial growth factor (VEGF) [60], insulin-like growth factor-1 (IGF-1) [61], platelet-derived growth factor (PDGF) [62], basic fibroblast growth factor (bFGF) [63], members of the transforming growth factor β (TGF-β) superfamily [64], Interleukin-6 (IL-6) [65,66,67], and connective tissue growth factor (CTGF) [49] have also been characterized in vivo and in vitro. They are up-regulated during the different stages of the healing process, resulting in increased cellularity and tissue volume [33].

TGF-β (isoforms TGF-β1, -2, and -3), and IGF-1 interfere at all stages of tendon healing stimulating inflammatory cell migration, proliferation of fibroblasts and other cells at the injury site, collagen, and ECM production [42]. It is well documented that the TGF-β activation pathway in response to injury is associated with scar formation and fibrous adhesion formation, and the suppression of the TGF-β1 signaling pathway enhances tendon healing in a rat model [68]. The three isoforms of TGF-β present different temporal patterns of expression over the course of tendon healing [69], suggesting that more detailed studies are needed in order to improve the outcomes of TGF-β applications in tendon healing.

Bone morphogenetic proteins (BMPs) are members of the TGF-β superfamily and play important roles in tendon healing. BMP-12 gene transfer in tendon cells increased the tensile strength and stiffness of lacerated tendons [70].

PDGF is also essential for tendon healing. Its administration in rat patella tendons increased the mechanical properties and tissue remodeling when delivered at a late stage after injury [71]. PDGF up-regulated tendon cell growth, collagen production, and ECM remodeling in vitro, but, according to recent work, PDGF may favor a trans-differentiation effect in tenocytes in culture [72].

Platelet-rich plasma containing high growth factor concentrations, among them tendino-inductive factors, gives promising therapeutic effects in vitro and in pre-clinical studies when delivered at the site of injury [73,74].

Biomaterials have been developed extensively to deliver growth factors to the site of injury. Understanding of scaffold design and manufacturing has been accumulated to allow for growth factors to be incorporated into the ECM or immobilized on its surface. In parallel, numerous studies have demonstrated the sensitivity of MSCs towards pro-tenogenic growth factors [29]. New techniques combining stem cells seeded on to scaffolds impregnated with growth factors could stimulate and guide tendon regeneration through the slow diffusion of biomolecules. Hydrogels have been explored to retain bioactive molecules to develop engineered tendon substitutes [75]. The use of a tenogenic differentiation medium (containing BMP-14, also known as growth and differentiation factor-5 (GDF-5)), was recently shown to enhance tendon-like matrix production from ASCs that are seeded on to poly(l/d)lactide (PLA) copolymer filament [76]. These authors reported a similar elastic modulus in bioengineered tissue and in native Achilles tendons.

##### Mechanical Stimulation

Tendons are subject to loads during movement, and are thus permanently under the effects of mechanical strains of different natures. It has been highlighted that application of physiological loads is necessary for maintaining tendon homeostasis, as well as for preventing excessive degradation of the ECM [77,78,79]. As a result, tendons are then in a continuous process of remodeling, adapting their metabolism, and structure [80]. These adaptations are made possible by the presence of cells in tendons. Fibroblasts have demonstrated their mechanosensitivity by proliferating [81] and producing collagen [82] when stretched through activation and/or the effects of a number of growth factors (details above). It has also been shown that mechanical force drives the development of tendons during embryogenesis [83]. In addition to growth factors, mechanical stimulation modulates cell differentiation, driving MSCs towards a tenocyte lineage [84]. In vitro studies outlined the importance of mechanical cues for the healing process of a lacerated tendon [85]. Thus, mechanical stimulation appears to be necessary for achieving correct tendon reconstruction by means of TE. Current strategies apply cyclic strain to achieve this goal, with a wide range of strains, frequencies, and rest periods [35,38,39,45,54,86,87].

#### 2.3.3. Materials

##### Biological Origin

Tendon composition and structure are mostly driven by type I collagen. For this reason, most research has focused on collagen alone or mixed with other molecules, such as proteoglycans as a support for tendon tissue engineering [88]. Different strategies have been explored to produce the ideal collagen-based scaffold, such as sponges [38,39,51,55,87,89], extruded collagen fibers [52,53,90], or electrochemically-aligned collagen [33,34,91], all being suitable for tendon reconstruction. In this review, simple films or collagen coatings are not presented because their inner mechanical properties are not relevant for TE applications.

Due to its rapid degradability, cost issues, and poor mechanical properties, alternatives to collagen for tendon reconstruction have appeared, including silk fibroin, one of two components synthesized by *Bombyx mori* silkworms during cocoon production [92]. With a fibrous nature, silk fibroin is a material with biocompatibility, low immunogenicity, and remarkable tensile strength as its main properties [93]. Silk fibroin has therefore been widely used for biomedical applications [94], such as silk yarns [95], knitted scaffolds [37,96,97], or electrospun materials [98].

More recently, decellularized matrices from tendons or other tissue origins were proposed as the “perfect” scaffold as they preserve biochemical composition, offering cells a full biomimetic environment. The chemical treatments performed to effectively remove donor cells may cause an inflammatory response when implanted into the host [99]. Of these chemical treatments, detergents, such as sodium dodecyl sulfate (SDS), 4-ocylphenol polyethoxylate (Triton X-100), or tri(n-butyl)phosphate (TnBP) are the most appropriate for fully removing cells from the tissue. Tendons from a wide range of species, including humans, rabbits, dogs, pigs, equines, rats, chickens, or bovines have been tested in order to find the best way to remove cells and to provide the suitable environment for tendon tissue engineering [100].

##### Synthetic Material

Synthetic polymers are very attractive candidates for TE as their material properties are typically more flexible than those of natural materials. Synthetic constructs present tunable and reproducible mechanical and chemical properties, they are relatively inexpensive to produce [73] and easy to mold into a variety of forms—meshes, foams, hydrogels, and electrospun. They can be non-toxic [101], and in many cases, processed under mild conditions that are compatible with cells [74,102,103].

Varied approaches have been deployed to generate scaffolds, such as electrospinning [35,45,46,54,104,105,106,107], yarns [35,107,108], knitting [36,37,97,109], and 3D printing [110], using a wide range of synthetic polymers such as poly (-caprolactone)(PCL) [35,111], poly-l-lactic acid (PLLA) [30,112], poly (lactic-co-glycolic) acid (PLGA) [105,106,113], or poly urethanes (PUs) [45,46,114].

##### Hybrid Material

Biologic-derived scaffolds have the advantage of being biocompatible and bioactive, recognized by cells, and favoring cell adhesion, migration, and proliferation. However, their rapid degradability and their low mechanical properties might limit their use in tissue engineering [115]. On the other hand, synthetic materials usually present low bioactivity, but better mechanical properties and slower degradation.

Hybrid scaffolds are based on the synergistic effect between natural and synthetic materials. Usually, the biological compound tends to act as cells’ carrier, stimulating proliferation and migration over the support, while the synthetic one provides the construct with the stiffness needed to reach mechanical properties near the tendinous native tissue [100]. For tendon tissue engineering, such biohybrid scaffolds have been produced from mixture of collagen and polyesters [107].

### 2.4. From Biohybrid Tendon Design to Reconstructed Tissue’s Response

We now propose a review of the different scaffolds, the mechanical properties achieved by the biohybrid constructs, as well as both in vitro and in vivo outcomes. We sorted the papers referenced (Table 1, Table 2 and Table 3), according to increasing scaffold’s complexity.

#### 2.4.1. Macroporous Sponge

Collagen has been widely-used to produce three-dimensional sponges alone [116,117,118,119,120] or in combination with other molecules present in the tendon, such as glycosaminoglycans [38,39,87], to further mimic the rich nature of tendon ECM. In addition, these molecules support cell cultures due to their inherent biocompatibility.

Freeze-drying using ice-crystals as a porogen makes possible the formation of macroporous sponges, allowing for nutriment transport and cell penetration, the main requirements for building a new tissue [117]. The pore structure of sponge mirrors ice-crystal morphology. Generally, interconnected pores with a random (isotropic) configuration are obtained. Anisotropic sponges have been successfully produced by incorporating a directional solidification step into a conventional freeze-drying process.

The group of Harley produced collagen-chondroitin sulfate anisotropic sponges placing the solution in a cold mold prior to sublimation to direct pore formation [38]. Several parameters affected the final pore size and the density of the macroporous sponges, such as solute concentrations or the freeze temperature (−10, −40 and −60 °C): the lower the temperature, the larger the pores’ diameter (243, 152 and 55 μm, respectively). Grier et al. (2017) increased the scaffold’s density using a cross-linking treatment [55].

In general, sponges have weak mechanical properties (an elastic modulus in the range of 1 kPa), but have nevertheless been used in tendon tissue engineering.

When cultured over anisotropic sponges with oriented pore distribution [38], horse tenocytes presented enhanced proliferation, metabolic activity, and alignment when compared to isotropic sponges. Larger pores (>150 μm) also enhanced cell proliferation and metabolic activity as compared to smaller ones [51]. In contrast, differentiation assessed by up-regulation of tendon-related markers (COL1, COL3, COMP, and DCN) was promoted on sponges with the smaller pores and high cross-linking densities [87].

Butler’s group has focused on the effect of mechanical stimulation on cell activity. For their studies, they worked with isotropic porous freeze-dried type I collagen sponges [38,39,87,120] with a mean porosity of 94% and pores with an average size of around 62 μm. Juncosa-Melvin et al. (2006) used these sponges to better understand the role of mechanical stimulation on the biomechanical properties of the final constructs [38]. Rabbit BMSCs were cultured for 12 days on the sponges with or without mechanical stimulation (8 h/day at 2.4% strain, once per minute). When stimulated, the constructs presented a linear stiffness and modulus 2.5 and 4 times higher than the non-stimulated ones. In the same study, the authors used those constructs to heal the patellar tendon in a rabbit model. Constructs that were stimulated prior to implantation presented better mechanical properties when compared to non-stimulated ones after 12 weeks of implantation. In another study, Nirmalanandhan et al. (2008) compared different sizes of sponge, long and short (51 vs. 11 mm of length), to better elucidate the importance of construct length in tendon repair [39]. After 14 days of culture, rabbit BMSCs that were cultured on the longest constructs presented a linear stiffness four times higher than that of short constructs (0.047 vs. 0.011 N/mm). Interestingly, for collagen-chondroitin sulfate constructs, a high level of COL1 and COL3 was found once stimulated at 2.4% of strain for 12 days with 3000 cycles per day when compared to collagen sponges [87].

#### 2.4.2. Collagen Extruded Fibers

As tendon presents an inherent alignment of collagen, the aim of recent studies has been to develop fibers that better mimic the native structure. Extrusion of type I collagen fibers has been successfully achieved, allowing for the production of fibers with a diameter varying from 10 to 2000 µm [121,122]. This fibrillogenesis is generally achieved by extruding a solution of acidic collagen over a gelation bath to shift acid pH to neutral [123].

To avoid rapid degradation, extruded fibers are generally reticulated with a combination of treatments, such as glutaraldheyde, cyanamide, carbodiimide, and dehydrothermal [124]. As a result, the fibers’ physical properties depend on the original collagen preparation, the fiber bath formation, the cross-linking treatment and the diameter of the extruded tube. Zegoulis et al. (2009) were the first to compare the mechanical properties of fibers that are produced through extrusion, depending on the cross-linking treatment. For example, non-reticulated collagen extruded fibers presented a fiber diameter of 300 µm and a maximum stress of 3 MPa, while after treatment with genipin, fibers of the same diameter reached a maximum stress of 7 MPa [124].

In a recent study, Enea et al. (2011) compared two methods (EDC or EDC/ethylene-glycol-diglycidyl-ether (EDGE)) to produce reticulated fibers [52]. EDC treatment resulted in softer and smaller fibers (stress at failure of 4.6 MPa; strain at failure 23.2%; modulus 19.3 MPa). EDC/EDGE resulted in stiffer ones (stress at failure 10.5 MPa; strain at failure 23.1%; modulus 46.2 MPa).

Although the cross-linking process provided better mechanical properties and degradation resistance, the reticulated fibers may present a lack of biocompatibility [52,53,125].

After 14 days of culture over the fibers, sheep tenocytes failed on cell colonization, proliferation, and collagen production on EDC/EDGE stiffer fibers when compared to the softer EDC ones [52]. Similarly, Ahmad et al. (2015) compared the effect on biomechanics and biocompatibility of different concentrations of two cross-linking agents, EDC and NHS [125]. While the agents’ concentration did not provide any significant effect on the mechanical properties of the fibers, the highest agent concentration resulted in less cell adherence and proliferation.

Following the in vitro study, Enea et al. (2013) used an open array of multiple fibers of extruded collagen to replace the patellar tendon in an ovine model [53]. After six months, EDC implants presented better integration and tissue ingrowth when compared to EDC/EDGE and higher stress to failure (4 vs. 1 MPa). These results highlight the need for the development of the correct cross-linking methods to better provide a biocompatible environment.

In addition, one can notice that most works have been carried out on single fiber experiments and there is still a lack of biological characterization in the presence of cells (differentiation, collagen synthesis). Further studies need to be performed with more complex structures, such as yarns, threads, or knitting scaffolds with collagen fibers.

#### 2.4.3. Electrochemically-Aligned Collagen (ELAC) Fibers

The Akkus team developed electrochemically-aligned type I collagen fibers (ELAC fibers) [33,34,126,127,128,129,130]. In the presence of an electric current (20VDC) produced by parallel electrodes, collagen molecules aligned at the isoelectric point, allowing for the production of collagen-aligned threads with a variable fiber diameter (50–400 µm) [126]. When reticulated with genipin, those ELAC threads showed mechanical properties in the range of those that are found on native tendons, with an ultimate tensile stress of 108 MPa, an ultimate failure strain of 13%, and a Young’s modulus of 890 MPa, showing the potential ELAC fibers have as carriers for tendon tissue engineering [129].

Kishore et al. (2012) compared ELAC threads (50–100 µm in diameter) with random collagen threads to better elucidate the influence of collagen alignment on human MSCs [33]. Interestingly, the cells adhered easily in ELAC threads when compared to random ones, but proliferation was higher in random than in ELAC threads. After 14 days, cells that were cultured over ELAC threads presented a spindle-shaped fibroblastic morphology and presented enhanced tendon early (scleraxis) and late (TNMD) differentiation markers after 3 or 14 days. On the other hand, cells cultured on random threads presented a random morphology and less tendon-related marker expression. The alignment of collagen threads is enough to produce tenogenic differentiation in the absence of any differentiation factors.

In another study, Younesi et al. (2014) showed the possibility of producing 3D bio-textiles with ELAC threads [34]. ELAC yarns (triple thread) were woven in a robust and porous scaffold (81% of porosity). This 3D configuration provided upgraded mechanical properties and a tendon characteristic-compliant toe-region when stretched. Further in vivo and in vitro studies need to be performed with these structures in order to confirm the trend and to ensure the promising results of ELAC threads as a strategy for full tendon replacement.

#### 2.4.4. Electrospun Scaffolds

##### Scaffold Structure and Mechanical Properties

Electrospinning leads to the production of fibers that mimic the ECM and therefore create a suitable environment for cell development [131]. There are a remarkable number of parameters that influence the structure of the final scaffold, such as the nature and concentration of the polymer and solvent, but also the form of the collector, conductivity, and displacement (static or rotating) [132]. The major materials that are employed in electrospinning techniques for further tendon engineering applications are polyhydroxyesters, such as PLLA [30], PLGA [105], or PCL [35] alone or combined [47], polyurethanes [45,46], and natural polymeric biomaterials, such as silk fibroin [133,134]. Generally, the fibers produced can thus be randomly deposited or aligned [30,46,47,105], flat, or three-dimensionally structured [35,135].

According to native structure, fiber alignment appears to be a target for mimicking the organization of collagen fibers in tendons. Moffat et al. (2009) produced PLGA random and aligned fibers using a rotating ground collector [105]. When the collector speed was high (20 m/s), the resulting scaffolds were composed of aligned fibers. The elastic modulus of aligned fibers was three times higher than random fibers (341 vs. 107 MPa). In another study, Yin et al. (2010) produced PLLA-aligned fibers using a rotator mandrel turning at 4000 rpm [30]. The mechanical properties of the aligned scaffolds were also enhanced with stiffness and modulus 46 and 36 times higher, respectively, when compared to random materials. As collagen fibers have a crimp-like structure of a variable range of wavelengths (between 45 to 65 µm) and amplitude of 5 to 10 µm [54], further studies have investigated the production of crimped scaffolds [136] and their role in promoting tendon-like tissues. To produce those fibers, Surrao et al. (2012) electrospun PLDLLA into a rotating wire mandrel made by two circular pieces allowing for the production of aligned fibers [54]. Once the final material was placed in a solution with a temperature 10 °C above the glass-transition (T*g*), the crimp patterns appeared as a result of the release of the energy stored during collection. This process made it possible to create a final electrospun scaffold made pf fibers with a diameter of 0.88 μm and a crimp amplitude and wavelength of 5.2 and 46 μm, respectively. The final modulus of the crimp scaffold was 3 ± 0.3 MPa.

Electrospinning is also a highly adaptable technique that allows for the production of a fibrous micron to sub-micron matrix. In the literature, one can find fibers from 40 to 2000 nm [137]. Erisken et al. (2013) produced PLGA fibers with diameters of 320 nm, 680 nm, and 1800 nm by modifying the polymer concentration [106]. Improved modulus and reduced ductility were found with the highest diameter fibers. In a similar study, Cardwell et al. (2014) synthesized different electrospun poly (esterurethane urea) (PEUUR) scaffolds with fiber sizes of <1 µm, 1–2 µm, and >2 µm aligned or random [46].

Although a thin layer of an electrospun material is very porous, the high packing density of such scaffolds prevents the correct colonization of cells through the material. In addition, when present as a fibrous sheet, electrospinning cannot be considered as a 3D environment. For these reasons, some researchers have been working on modified electrospun set-up devices in order to produce improved scaffolds with high porosity and a 3D structure. Sacrificial fibers [138], air-gap [139], water bath collection [107,140,141], or twisted electrospinning to make yarns [35,107,141], have appeared to be a promising solution to confer electrospun scaffolds a superior ultrastructure.

Bosworth et al. (2014) proposed three-dimensional electrospun yarns by continuous strands of twisted aligned PCL fibers resulting in yarns with a final diameter of ~150–200 μm [35]. When compared to a two-dimensional (2D) aligned scaffold, 3D yarns presented a higher ultimate tensile strength and Young Modulus (5 and 14 MPa vs. 1 and 5 MPa). In another study, Xu et al. (2013) produced electrospun yarns through a modified water bath collection system [107]. First, P(LLA-CL) and type I collagen fibers were collected in a water basin with a hole in its bottom. As water was continuously drained, the collection system created a vortex flow, producing twisted yarns, and then collected the yarns in a rotating drum. The final yarns were made of aligned fibers with a diameter of 640 nm. When compared to it homologous 2D aligned electrospun scaffold, nanofibrous yarns presented a lower Young’s Modulus (2 vs. 4.5 MPa) and lower tensile strength (4 vs. 6 MPa), but higher break at elongation (150% vs. 250%).

In the following section, the interactions between cells and scaffold structures, such as fiber distribution (aligned vs. random, and fiber size), or 2D vs. 3D structure will be presented.

##### Biological Response

To analyze the effect of scaffold alignment, Moffat et al. (2009) cultured human rotator cuff fibroblasts on PLGA scaffolds with different structures (random vs. aligned) [105]. After 14 days of culture, no differences in cell proliferation were observed. The aligned fiber scaffolds maintained their mechanical properties longer than the random ones in culture, and fiber alignment appeared to be the main contact guidance to make cell attachment and alignment possible along the fiber axis. In a similar study, Yin et al. (2010) compared the effect of PLLA fiber alignment on hTSPCs [30]. When cultured over aligned scaffolds, hTSPCS showed a spindle-shaped morphology, a classic fibroblastic phenotype. In addition, cells that were cultured on aligned fiber scaffolds presented tendon up-regulated expression and matrix deposition (collagen) and resisted bone induction when compared with random scaffolds. When the same scaffolds were implanted in an ectopic murine model, aligned morphology and collagen synthesis were also found to be enhanced when fibers were aligned.

The effects of fiber diameter on cell activities have been investigated. In a study by Erisken et al. (2013) human rotator cuff fibroblasts were cultured over scaffolds of PLGA with different fiber sizes [106]. In contact with the different mats, cells presented high production of a tendon-like matrix (COL and GAGs) in nano-fibrous scaffolds, but high tendon-related marker expression (COL1, COL3, and TNMD) in larger fiber scaffolds after 28 days of culture. In a similar study, Cardwell et al. (2014) were interested in the effect of fiber diameter on the differentiation of C3H10T1/2 cells into tendon/ligament lineage [46]. After nine days of culture, cells achieved tendon/ligament-differentiation and produced more collagen on larger fibers, regardless of fiber alignment. Taken together, it seems that small, nano-scale random fibers provide a cell environment similar to that found in the inflammatory phase of the tendon healing process, promoting the synthesis of the ECM and cell proliferation, while larger aligned fibers mimic the normal structure of collagen in tendon, maintaining the tendon cell phenotype. This could explain why larger fibers promote high levels of tendon-related gene expression, ensuring the maintenance of the fibroblast phenotype [142].

Bosworth et al. (2013) compared the effect of scaffold structure on cell behavior [143]. When seeded with equine tendon fibroblasts, the cells presented an alignment through the direction of the fibers and an augmented proliferation over time (14 days), however, proliferation was less pronounced on yarns due to the smaller surface area when compared to flat 2D electrospun scaffolds. In a similar study, Xu et al. (2013) compared cell activity over P(LLA-CL)/collagen yarns and its 2D equivalent [107]. After 14 days of culture, primary tendon cells that were cultured on yarns presented enhanced expression of tendon-related ECM genes (*COL1*, *Decorin*, *TNC* and *Biglycan*), proliferation and colonization compared to 2D-aligned scaffolds.

##### Effect of Mechanical Stimulation

Independently of fiber diameter or alignment, mechanical stimulation was suggested to induce tendon-like cell responses with up-regulation of the expression of tendon-specific markers and ECM production both in vivo and in vitro [28,29]. Cardwell et al. (2015) studied the effect of both fiber diameter and mechanical stimulation (static or dynamic load) on cell activity [45]. These authors plated C3H10T1/2 cells on PEUUR fibers with different sizes (600 vs. 1750 nm) under static (50 mN) or a dynamic load (4% cyclic strain for 30 min at 0.25 Hz daily). After three days of culture, no significant changes in COL1, COL3, DCN, or cell alignment was found. Moreover, cells in contact with larger fibers under static load presented elevated levels of TNC and TNMD, suggesting that the fiber diameter and the mechanical environment may alter cell activity.

For Jha et al. (2011), when bovine fibroblasts were cultured over crimp patterns and submitted to mechanical stimulation above the unfolding region of the crimp structures, cells produced more tendon/ligament-like tissue (collagen and proteoglycans), and interestingly, crimped scaffolds retained their mechanical properties over time [139]. In 3D nanofibrous electrospun yarns, Bosworth et al. (2014) investigated the response of human mesenchymal stem cells (hMSC) when cultured under dynamic loading [35]. During the experiment, electrospun yarns were stimulated for 7 or 21 days, once per day at 5% of elongation and 1 Hz. When submitted to dynamic load, the cells underwent morphological changes and an up-regulation of tendon-related markers (COL1, COL3, TNC, FN). Under dynamic conditions, the cells presented on the outer circumference of the yarns, were more round and the cell layer was thicker when compared to the static conditions. Xu et al. (2014) also investigated the effect of mechanical stimulation over electrospun nanofibrous yarns [141]. After 14 days under dynamic loading (4% elongation at 0.5 Hz, 2 h/day), rabbit TDSCs presented an aligned morphology in both static or dynamic cultures, but major proliferation and tendon ECM production (COL1, COL3, TNC) and enhanced expression of tendon-related markers (COL1, COL3, decorin, TNC, biglycan) under dynamic load. After twelve weeks of implantation in a full-size defect in a rabbit model, biohybrid scaffolds that were prepared under dynamic conditions presented better cell alignment, ECM synthesis, and mechanical properties than those that were prepared under static culture.

On the basis of this literature review, it is possible to say that there is still no consensus on the effect of mechanical stimuli on cell differentiation and production of ECM. This might be due to the absence of consensus regarding the frequency and amplitude of the stimulation to apply.

#### 2.4.5. Knitted Scaffolds

The application of textiles techniques has been widely-used for tissue engineering as it offers the possibility of creating complex hierarchical 3D structures with tailored mechanical properties similar to native tissues [144]. Knitting offers the possibility of creating 3D structures made of interconnected loops of yarns or threads [109] that determine both their mechanical properties and their porosity [37]. To create these structures, a combination of biological and/or synthetic materials, such as silk or PLGA, has been tested [36,37,97,145]. Combined with electrospinning or sponges, this makes it possible to produce multi-hierarchical structures that mimic the nature of the rich tendon ECM.

Sahoo et al. (2006) produced a combined nano-micro fibrous knitted scaffold with the combination of PLGA micro fibers (yarns of 25 μm) and electrospun PLGA nano fibers (300–900 nm) [36]. The final combined construct presented pore size from 2 to 50 μm, an initial failure load of 56.3 N and an initial elastic stiffness of 5.80 N/mm. After 14 days of culture, BMSCs showed increased proliferation, collagen production, and up-regulation of tendon related-markers (COL1, decorin, and biglycan) when compared to the PLGA knitted control without electrospun fibers.

In another study, Liu et al. (2008) developed a knitted silk scaffold resulting from interconnected loops with a pore size of 1 mm and good mechanical properties, with a maximum tensile load of 252 N and a stiffness of 40 N/mm [37]. One of the main problems of knitted scaffolds is finding the right way to load the cells. To improve cell loading and proliferation, these authors placed the knitted construct in a silk solution. Once freeze-dried, this made it possible to produce a combined scaffold with final pore sizes from 20 to 100 μm. The mechanical properties of this combined scaffold were similar to those of simple knitting, with a maximum tensile load of 255 N and a stiffness of 45 N/m. After 14 days of culture, human BMSCs showed enhanced proliferation and ECM production (COL1, COL3 and GAGs) in combined scaffolds compared to simple silk knitted scaffolds. In a similar study, Zheng et al. (2017) studied the effect of the pore direction of the collagen macroporous sponge on knitted scaffolds [97]. Twelve silk yarns (pore size of 1 × 1 mm) were placed in a type I collagen solution. Unidirectional freezing made it possible to produce aligned pores, while random sponges were made by classic freeze-drying. The final pore size of aligned sponges (110 µm) was smaller than that of the random ones. After seven days of culture, rabbit TSCPs presented the same attachment, spreading and proliferation in both constructs while ECM deposition was aligned into knitting constructs combined with aligned pores, and random constructs with random pores. In a tendon repair model in rabbits, rectangular defects (10 × 5 mm) in the rotator cuff tendon were filled with random or aligned constructs for 4, 8, and 12 weeks. After 12 weeks, the regenerative tissue was more organized and with more ovoid cells, and collagen fibers were larger and denser in aligned constructs when compared to random constructs, similar to the results found in normal tendons.

## 3. Skeletal Muscle

### 3.1. Skeletal Muscle’s Composition and Structure

Skeletal muscle is a dynamic tissue that is responsible for voluntary movement, postural maintenance, and soft tissue support, through the conversion of chemical energy into mechanical force applied to bone via tendinous tissue. Skeletal muscle is the most abundant tissue in the human body, representing approximately 40% of body mass [146]. The architecture of skeletal muscle is characterized by a highly ordered arrangement of muscle fibers associated with connective tissue [147] (Figure 2). The cellular structural unit of skeletal muscle is the myofiber. A myofiber is a multinucleated single muscle cell, which ranges from approximately 20–100 µm in diameter. Myofibers are arranged in parallel, with length ranging from a couple of mm to several tens of mm depending on the muscle [148]. Myofibers are wrapped in a fibrous ECM, the endomysium, and bundled in fascicles, each of which is supported by the perimysium (Figure 2). There are thus three fibrous layers of connective tissue in skeletal muscle, i.e., the endo-, peri-, and epi-mysium, the latter enveloping the muscle, and supporting the structural and functional continuity of the muscle-tendon junction. They are composed of collagen (types I and III, mainly) and proteoglycans mostly from the family of small leucine-rich proteoglycans (SLRPs). Decorin is the major proteoglycan in the perimysium [149].

The differentiation of skeletal muscle cells is stimulated by a contact-dependent process. Myofibers are thus formed when undifferentiated muscle cells (myoblasts) fuse together to form elongated, multinucleated myotubes, gathering nuclei in a central position. As the myotubes mature to form myofibers, the nuclei adopt positions near the plasma membrane at the cell periphery [150]. At the ultrastructural level, the major components of myofibers are myofibrils, which represent the molecular machinery that is capable of controlling muscle stretching thanks to a sliding movement between the thin, actin filaments, and the thick myosin ones. Actin and myosin proteins represent approximately 70% of the total protein content of a single fiber [151] and are the main component of sarcomeres, the smallest chain of contractile units (approximately 2.3 μm long). Each myofibril is composed of hundreds of sarcomeres in series. It should be noted that skeletal muscle fibers differ in their phenotypes depending on their myosin heavy chain isoform, which results in differences in twitch speed. Type I fibers express slow-twitch myosin heavy chain (MyHC) isoforms and are suited for endurance while type II fibers express fast-twitch MyHCs that are suited for short and high intensity work [152].

Collinsworth et al. (2002) etablished that skeletal muscle cells exhibited viscoelastic behavior that changed during differentiation: the apparent elastic modulus increased from 11.5 ±1.3 kPa for undifferentiated myoblasts to 45.3 ± 4.0 kPa after eight days of differentiation [153].

As well evidenced by Heinemeier et al. (2013), skeletal muscle is a very physiologically active tissue. The high rate of tissue turnover leads to continuous renewal of core muscle. This remarkable capacity for regeneration found in skeletal muscle is made possible through the activation of resident multipotent cells to compensate for muscle tissue turnover or in response to injury [154,155]. The most important cells implicated in the regenerative response of muscles are satellite cells. They are an quiescent population of resident muscle progenitor stem cells, which, in response to injury, are activated and migrate to the defect site, expand, and undergo myogenic differentiation or self-renewing of the satellite cell pool [156].

During muscle regeneration, satellite cell behavior is regulated through a cascade of complex signaling pathways controlled by intrinsic factors within satellite cells, as well as extrinsic factors that compose the muscle stem cell niche/microenvironment [157]. Behind these major muscle resident progenitors, fibro/adipogenic progenitors (FAP) have also been described as promoting muscle regeneration through ECM deposition and promyogenic factor secretion. In the case of chronic muscle injuries, the controlled response of FAP may be unbalanced in favor of excessive ECM deposition, leading to fibrosis and impaired muscle regeneration efficiency [158].

### 3.2. Muscle Injuries and Healing

Skeletal muscle injuries typically result from traumatic incidents, such as contusions and strains during sports activities, as well as trauma due to accidents or surgical resection of tumors, and are designated as volumetric muscle losses (VMLs). Approximately 35–55% of all sports injuries involve skeletal muscle damage to the myofibers and/or connective tissue [159]. Furthermore, about 5.8 million reconstructive surgical procedures are performed annually as a result of cancer ablation or road traffic accidents [160]. The detailed healing process of skeletal muscle following trauma has already been well described elsewhere [148,161,162,163]. Briefly, the healing process is composed of three phases: destruction, repair, and remodeling. During the destruction phase, after necrosis of the ruptured myofibers, the propagation of this necrosis is stopped within a couple of hours by a contraction band in the shelter of which the rupture is sealed by a sarcolemma. The broken myofibers contract and the gap between the stumps is filled by a hematoma, meaning that an inflammatory cell reaction occurs. The repair phase starts with phagocytosis of the necrosis surface by blood-derived monocytes. The myogenic process is then activated by activation of the satellite cells. This activation leads to differentiation into myoblasts, followed by a proliferation stage over 24 h, which contributes to the formation of myoblasts. Finally, these myoblasts fuse to form myotubes within a couple of days. After 5–6 days, the necrotic part is replaced by the regenerated myofibers. Revascularization of the injured site occurs three days after the injury with the formation of angiogenic capillary sprouts. The last repair phase, the remodeling phase, is characterized by the maturation of the newly regenerated myofibers, i.e., a maturation of the contractile material and attachment of the ends of the regenerated myofibers to the intervening scar by a newly-formed musculo-tendinous junction.

#### 3.2.1. Grafts

Critical-sized tissue loss of muscle mass (more than 20%) impairs endogenous repair mechanisms [164]. In these cases, the gold standard procedure is most often achieved by autologous tissue transfer (graft) from an uninjured site in the patient [165], such as the muscle flap transfer [166]. Although frequently successful, harvesting soft tissue from the patient creates new defects and the possibility of increased morbidity. Allografts are used to bypass the drawbacks of autografts, but they are beset by limitations in supply, tissue condition at the time of transplant, and concerns over immunogenicity, morbidity, and cost [167].

#### 3.2.2. Cell Therapy

Cells therapies have been investigated when the regenerative capacity of the skeletal muscle is partly depleted, as in severe myopathies, such as Duchenne or Becker muscular dystrophy. This therapeutic strategy relies on the delivery of myogenic precursors or stem cells to the muscle tissue to improve regeneration and tissue repair thanks to structural and functional integration in the host tissue. It requires a suitable cell population, which is capable of proliferating in vitro to generate sufficient cell quantities for transplantation. Of the cellular candidates, satellite cells, primary myoblasts, fibro-adipogenic progenitors (FAP), and human pluripotent stem cells are considered as promising cell sources thanks to their high regenerative potential in situ or their unlimited proliferative capability.

Despite the potential efficacy of cell-based therapies in muscle regeneration, the poor outcomes of preclinical and clinical trials identified a number of issues [168]. The injected cells face a harsh environment, not only because of the inflammatory response to the muscle injury, but also due to the injection process itself. Intramuscular injections can further damage the tissue, while going through the systemic system, the cells may be unable to attain the injured muscle and instead engraft on to other tissues or organs [139]. Thus, regardless of the approach used, most cells fail to survive a few hours after injection. The cell culture conditions used to expand the cell before the transplantation step need to be improved to maintain the “stemness” or myogenicity characteristics of cells [169]. Interesting studies have shown the influence of substrate physical properties on skeletal cell differentiation. The substrate, on which cells are cultivated, with compliance and elasticity cues mimicking those of the muscle cell micro-environment, may be a regulator for myogenicity [170,171]. Some of the problems that are associated with cell therapies may be fixed by adopting an approach that includes biomaterials as a niche for cells, leading to muscle tissue-engineering strategies.

### 3.3. Skeletal Muscle Tissue Engineering

In this part, we selected the publications of interest, as described in Section 2.3. However, in contrast with tendon, skeletal muscle’s properties (and specifically contractility) are mainly driven by cell behavior. The main approaches that can be found in the literature in muscle TE thus focus more on the end behavior of the cells after culture in a scaffold. The mechanical and biological outcomes investigated are thus quite different to those observed in tendon TE. The major biological issues concern myotube formation from myocytes, and contractility properties. The mechanical properties of a biohybrid construct are poorly documented, with the scaffold appearing mostly as a guide for cell organization and differentiation. In addition, one can point out that muscle tissue engineering is a recent approach, with the first papers appearing in 2005.

The publications of interest are presented in two tables. The first (Table 4) deals with general details of the Materials and Methods part, the second (Table 5) reports in vitro outcomes. Due to a lack of information, there is no table summarizing in vivo results.

#### 3.3.1. Cells

The choice of an appropriate cellular source is fundamental for generating functional muscle in vitro. Fishman et al. (2013) established a non-exhaustive list of criteria that cells should meet to be suitable candidates for muscle engineering [172]. According to the literature data (Table 4), four cell types are predominantly employed in muscle engineering: the mouse C2C12 myoblast cell line [173,174,175,176,177,178,179,180,181,182,183,184,185,186,187,188,189,190,191,192,193,194,195,196,197,198], primary myoblast-derived satellite cells (SCs) [175,199,200], primary myoblast from different species [181,201,202,203,204], and mesenchymal stem cells (MSCs) [177,205]. SCs are an appealing solution as they are relatively easy to isolate and are also the direct precursor of myoblasts. Unfortunately, SCs maintained in vitro suffer a severe reduction in their ability to produce myofibers, and a decrease in their proliferative capacity [206]. The C2C12 cell line manages to decrease the variability of primary cell isolation. In addition, using the C2C12 cell line for muscle engineering studies makes possible an objective comparative analysis with works that are published in skeletal muscle bioengineering as it mainly uses this cell type [207].

All of these four cell types are helpful for preliminary design, but there is, to our knowledge, no attempt to cultivate myoblasts or satellite cells of human origin in scaffolds for TE yet.

#### 3.3.2. Modulation of the Environment

**Figure 5:**
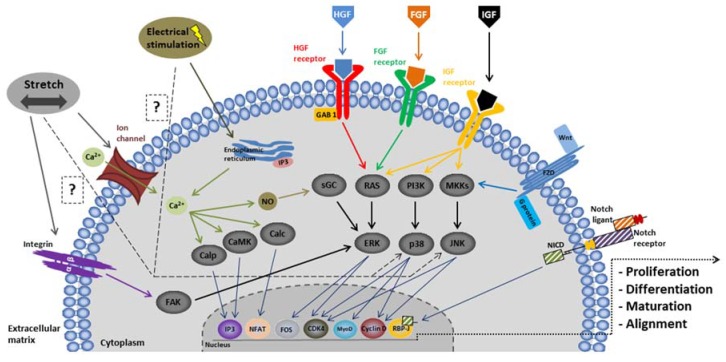
Schematic representation of skeletal muscle cell mechanotransduction: chemical signals are initiated by growth factors such as insulin-like growth factor (IGF), Hepatocyte growth factor (HGF), and fibroblast growth factor (FGF) binding to their respective receptors to trigger RAS, phosphatidylinositol-3-kinase (PI3K), and McKusick-Kaufman syndrome (MKKs) signaling cascades and activate Extracellular signal-regulated kinases (ERK), mitogen-activated protein kinases (p38), and c-Jun NH2-terminal kinases (JNK) pathways, respectively [233,234,235]. Electrical stimulation induces calcium release from the endoplasmic reticulum [236]. Calcium can act by activating either ERK [237] or calp, camk and calc [238,239,240]. Mechanical stretching signals involve the transmembrane protein integrin and the calcium ion channel [241]. Activating integrin triggers the FAK signaling pathway. Electrical and mechanical stimulations are also likely to activate the JNK and p38 pathways. Other pathways may be involved, such as wnt/frizzled and notch. All these signaling pathways up-regulate the expression of some of the genes responsible for skeletal muscle progenitor development.

Functional muscle formation is an intriguing and highly complex process that requires features, such as cell differentiation and maturation [208]. As shown in Figure 5, several intracellular pathways are responsible for enhancing proliferation and differentiation expression of cell genes during muscle development [209]. The effects of a wide variety of chemical and/or physical factors on muscle cell progenitor cultures have been investigated extensively. Many previous studies have demonstrated the ability of chemical stimulation to induce muscle cells and differentiation by studying the effect of certain growth factors [210,211,212]. At the same time, many studies suggest the benefits of using physical factors because of their potential ability to accelerate growth and development in skeletal muscle engineering [213,214,215,216]. Electric and mechanical factors are the most commonly used in the literature. Electrical stimulation is of particular interest because of the indisputable role of the electrical cues issued by the central nervous system in the development of skeletal muscles in vivo [217]. The understanding of its effect and how to use it are increasingly controlled. The parameters of the electric field applied can be modulated, according to the type of response desired. It has been shown that depending on whether the regimen applied is direct or alternative, and depending on the voltage/intensity range, it accelerates sarcomere assembly, promoting cell proliferation, differentiation, and/or muscle cell alignment [173,183,192,194,199,200,202,218,219,220]. Some studies pointed out that electrical stimulation makes intracellular calcium and NO release possible [221]. Others showed that it acts via the activation of PI3K, p38 signaling pathways [222,223]. In parallel, mechanical stresses also play a role in muscle cell growth, differentiation, and function because of the contractile and elastic nature of skeletal muscle [224]. When cells grow on a scaffold, a variety of stretch regimes can be applied. Thus, by modulating the cycle, stretching elongation and duration, muscle cell changes and functionality can be modulated [171,174,175,176,185,203,225,226]. It seems that cell stretching induces the activation of FAK via integrin, leading to an increase in gene expression [227]. Other studies suggest that stretching may also influence the passage of calcium via the ion channels [228,229] and activate PI3K and p38 signaling pathways [230,231].

It has now been clearly shown that several signaling pathways can be modulated in order to control muscle cell development in tissue engineering. The most recent studies are based on cell culture methods while using a combination of chemical and physical stimulations. More importantly, there is growing evidence that a combination of chemical and physical stimulations in addition to surface topography and scaffold composition may be a solution for generating safe and functional muscle constructs in vitro [184,232]. However, the chronology of these different stimuli actions during the development of muscle cells in vivo remains unclear. It may be of particular interest to investigate not only a combination, but also successive different stimulations (chemical, mechanical, electrical).

IGF, insulin-like growth factor; HGF, Hepatocyte growth factor; FGF, fibroblast growth factor; PI3K, phosphatidylinositol-3-kinase; MKKs, McKusick-Kaufman syndrome; ERK, Extracellular signal-regulated kinases; p38, mitogen-activated protein kinases; JNK, c-Jun NH2-terminal kinases; sGC, soluble guanylyl cyclase; calp, calpain; calc, calcineurin; CaMK, Ca^2+^—calmodulin-dependent protein kinase.

#### 3.3.3. Materials

##### Biological Origin

The macromolecular composition and structure of protective sheets surrounding muscle fibers (e.g., sarcolemma, endomysium) are mostly driven by various types of collagen [173,174,176,199,200,242,243]. For this reason, collagen and gelatin have been widely used as materials for muscle tissue engineering [182,183,194,244]. Non-mammalian sources of naturally derived materials have also been explored to produce suitable scaffolds for muscle reconstruction, such as alginate [177,245], fibrin [175,202,246,247], or chitosan [205,248]. They have the capacity to be configured into various shapes, including film, hydrogel, and sponge. Some of these materials are responsive to fabrication methods, such as chemical modification to add cross-linkers [249], or specific functional groups to improve cell attachment [250], or mechanical properties [251], in order to obtain structural control similar to that of native muscle.

Recently, as with tendons, scaffolds that were derived from decellularized skeletal muscle may be the optimal biomimetic biomaterials for repairing large skeletal muscle defects. In the literature, implants of decellularized muscles have been reported with contrasting results. Lin et al. (2014) showed that the enzyme detergent method for removing cells from mouse skeletal muscle, made it possible to maintain the biomechanical properties at a level that was comparable to that of native tissue [252]. Several other authors did not observe any myoblast migration towards the scaffold in vivo [253,254]. More recently, Porzionate et al. (2015) performed a comparative analysis between different decellularization protocols on muscles from different species, and especially on human samples. The study evaluated the integration capacity of the decellularized scaffold in vivo. They observed good integration of the scaffold surrounding the native muscle structure and signs of neo-vascularization [255].

##### Synthetic Materials

Most of the synthetic polymers used for muscle tissue engineering scaffolds are manufactured from polyesters, which include poly(vinyl alcohol) (PVA) [198,205], (PGA) [256,257], poly(lactic acid) (PLA) [258,259], poly(caprolactone) (PCL) [190,191,260], and their copolymer poly[(lacticacid)-co-(glycolic acid)] (PLGA) [113,186,190,195,261,262]. These polymers are well characterized and have been approved by the Food and Drug Administration (FDA) for certain human uses [263]. They can be tailored into porous sponges, fibers, or microspheres for cell encapsulation [261]. PDMS (polydimethylsiloxane) [178,220], which is a type of silicone, is also used for other bio-microsystem applications. Although there are many applications in TE, their lack of biological cues for promoting desirable cell adhesion and responses may be a problem and requires specific coatings [178,220,264].

##### Hybrid Materials

Hybrid scaffolds consist of the combination of synthetic polymer and natural derived components, in an attempt to benefit from and exploit each asset. Natural components bring bioactivity, favorable environments for cell adhesion, and proliferation, along with remodeling properties, while synthetic materials can obtain the target mechanical properties. Although this type of approach is quite recent for muscle reconstruction, several configurations and combinations can be found in Table 4: PDMS and fibrin [247], PEG and fibrin [204], PLGA and collagen [195], PCL and collagen [265], and PCL and silk fibroin [196].

### 3.4. From Biohybrid Muscle Design to Reconstructed Tissue’s Response

#### 3.4.1. Films and Hydrogels

##### Effect of Scaffold Structure and Mechanical Properties on Biological Response

Of the materials used, collagen [173,174,176,188,199,200,203,213,243], fibrin [175,202,204,246,266], gelatin [182,183,194,267], alginate [177,245], and polymers, such as PLLA [180], PDMS [178,220], or PEG [196,268] generally functionalized or coated with adhesion peptides, are the most commonly found. To compensate for the mechanical weakness of hydrogels and their lack of conductive properties, which are useful in muscle tissue engineering [269,270], nanomaterials have often been added to the initial polymer. These include gold nanostructures [265,271], graphene [179,195,272], and carbon nanotubes [192,194,198,273,274]. The rationale for developing conductive polymers is the need for the transmission of the electrical impulse, which in turn may influence cell behavior, specifically for cardiac and skeletal muscle [275].

Natural polymers were first used in the form of simple coatings, to efficiently exploit the inherent capacity of cells to produce their own extracellular matrices and assemble themselves into organized and functional tissues. The gel-like structure and smooth aspect of the coating induce cells to proliferate and differentiate in a random orientation. To overcome this anarchic cell arrangement and favor myotube alignment, which is one of the most critical factors in skeletal muscle regeneration, Vandenburgh et al. (1988) anchored the gel between two fixed points acting as an artificial tendon. Mechanical tension between the anchor points promoted myofiber alignment and stimulated muscle growth [276].

Several studies outlined the role of film stiffness on myotube differentiation into the physiological striated state. The best results were obtained on materials with muscle tissue-like stiffness (elastic modulus around 10 ± 4 kPa) [170,277]. Baniasadi et al. (2016) worked on cross-linked-oxidized alginate/gelatin hydrogels and investigated the impact of mechanical properties and degradation rate on the behavior of cultured cells [177]. In order to contract, muscle fibers need to grow parallel [278] to one another with identical anisotropy [279]. This can be achieved using a film with a specific topography to induce this behavior via contact guidance [280].

Topographical nano- [281] or micro-patterning have thus been investigated in grooves [282], waves [178], or more complex configurations [283] to enhance rat satellite cells or C2C12 myoblast fusion thanks to alignment and myotube formation. This approach mainly applied 2D films on to which myoblasts were cultured as monolayers until the formation of mature myotubes. Then, the mature cell layer can be transfer into a 3D contruct hydrogel [247], in order to be transplanted into a rat model. Several studies have shed light on the effect of optimized surface features, such as groove depth [180], width [181], and periodicity [178] on the formation of longer, functional myotubes with striated structures and contractile behavior in vitro [284]. According to these authors, optimal depth varied between 1 to 2.5 µm for a width of 10 µm, with a periodicity of 6 mm. Bajaj et al. (2011) demonstrated that hybrid 30° patterned structures led to the best C2C12 cell differentiation, as assessed by myosin and nuclei staining, as well as the size and orientation of the resulting myotubes [220].

Hydrogels were also developed in 3D to embed/encapsulate the seeded cells. Costantini et al. (2016) prepared a chemically-modified gelatin hydrogel and demonstrated the positive impact of mechanical stiffness and geometrical confinement on myoblast culture. Their results showed a parallel orientation of cells cultured in the smallest hydrogel string structure. Interestingly, the highest amount of myotube formation was obtained in a 3D hydrogel with stiffness in the range of 3 kPa, when compared to hydrogels whose stiffness was closer to that of native tissues. They speculated that C2C12 cells, when cultured in a 3D environment, exhibit specific focal adhesion configurations that influence cell polarization and signaling pathways, which were not observed in 2D constructs [285].

In contrast, Cvetkovic et al. (2014) produced strips of cross-linked collagen and fibrin with very high elastic moduli from 200 to 400 kPa that they placed on a specific holding tool named “biobiot”. Despite the considerable stiffness of the material, cells aligned during gel compaction and formed myotubes, more specifically, under the effect of IGF added to the gel [286].

Hydrogels can be shaped as sponges, with an interpenetrating network structure favoring cell colonization within the 3D scaffold. For example, Bandyopadhyay et al. (2013) developed a biocompatible and biodegradable porous sponge that is made with poly(l-lactide-co-ε-caprolactone) copolymers using phase inversion [201]. This type of scaffold, which is characterized by a pore size of around 300 µm, supports adult human myoblast growth and differentiation into multinucleated myotubes in vitro and favors cell colonization in vivo in an ectopic rat model. Similarly, Kin et al. (2007) prepared cross-linked atelocollagen sponge using a freeze-drying technique (−80 °C), with pores in the range of 50–100 µm, and successful cell colonization of the scaffold was achieved in an ectopic rabbit model [243]. Although the hydrogel/sponge manufacturing process is relatively easy to implement, pore size and full interconnectivity remain difficult to control [287,288]. Another way of controlling 3D hydrogel porosity is to mold them into previously prepared PDMS structures that are designed by photolithography. In the study by Bian et al. (2012), primary muscle cells from rats were mixed with matrigel/fibrin gel to form an elongated hexagonal structure of various sizes. They demonstrated that the networks with the most elongated pores resulted in the best cell response in terms of alignment and contractility [204,278].

##### Effect of Electrical Stimulation on Biological Response

Recently, both Kasper et al. (2018) and Rangarajan et al. (2014) highlighted the attractive strategy of electrical stimulation for activating the signaling pathways that are presented in Figure 5 [289,290]. Hashimoto et al. (2012) demonstrated the effect of electric field on the differentiation and contraction of cultured C2C12 cells. More specifically, they showed that optimized parameters (1s pulse of 8V for three days) had a beneficial influence whereas higher electric stimulation damaged myocytes [173]. Serena et al. (2008) aimed partly to mimic neuronal activation by means of an adequate electrical field (pulse of 70 mV/cm for 3 ms). Applying this to muscle precursor cells (MPCs) cultured in 3D collagen scaffolds, they observed enhanced proliferation when compared to non-stimulated cultures. However, ten days after implantation in mice, cell number and distribution were no different in the two conditions [199]. Cvetkovic et al. (2014) subjected their constructs that were located on “biobots” to electrical stimulation (20 V, 1 to 4 Hz), representative of action potentials observed in vivo. They managed to coordinate the contraction of multiple myotubes in the artificial muscle strip [286]. In contrast, Stern-Straeter et al. (2005) focusing on the influence of electrical stimulation of primary myoblast cultures in a 3D degradable fibrin matrix, described the negative impact that is induced by their stimulation on the myogenic differentiation process, with a down-regulation of the transcription factor in the MRF-family [202]. Coordinating the electrical stimulation within the differentiation process of muscle progenitor cells is delicate and should not be introduced too early [200].

##### Effect of Mechanical Stimulation on Biological Response

A number of studies applied mechanical loading to cell-laden scaffolds in order to develop functional and structurally-biomimetic muscle constructs. Mechanical stimulation is another important factor during myogenesis [203,208], through the continuous passive tension applied to skeletal muscle by bone growth during both embryogenesis and neonatal development, as described in Figure 3. It also has a significant impact on the diameter of mature skeletal muscle fibers, as well as on cell numbers and myofiber composition [291].

Twenty years ago, Okano et al. (1997) described the impact of cyclic mechanical stretching (frequency: 60 Hz, amplitude: 5%, for four days) on encapsulated C2C12 myoblasts in a collagen type I gel, and reported an assembly of highly dense and oriented myotubes [176]. More recently, Powell et al. (2002) outlined that repetitive stretch/relaxation cycles applied to muscle cells suspended in collagen/Matrigel enhanced the diameter and area of myotubes by 12% and 40%, respectively, and increased the elasticity of the muscle construct, after eight days [203]. Pennisi et al. (2011) mobilized uniaxial or equibiaxial cyclic tensile strain (15% of stretch, 0.5 Hz) to induce assembly and differentiation in C2C12 skeletal myocytes seeded on to flexible-bottom plates precoated with collagen-I. The uniaxial strain resulted in a highly aligned array of cross-striated fibers, with the major axis of most cells aligned in a perpendicular manner in relation to the axis of the strain, and caused faster cell differentiation; on the other hand, equibiaxial strain did not induce any clear orientation and it displayed signs of membrane damage and impaired differentiation [174].

The mechanical stimulation of muscle constructs has not been systematically associated with an improved biological response, depending on the strain parameters used (duration, frequency, direction) [203]. For instance, Boonen et al. (2010) investigated the effects of a two day uniaxial ramp stretch (2%), followed by four days of uniaxial intermittent dynamic stretch (4%) at a frequency of 1Hz on the C2C12 or MPC cells in 2D or 3D constructs. They observed either no effect or a lowered effect on the maturation and differentiation of the cells [175]. There is thus not yet any consensus on the protocols to be applied to such constructs.

The simultaneous combination of mechanical forces and geometric constraints imposed by the substrate represents new models for understanding the mechanisms of cell response.

Ahmed et al. (2010) recently designed a flat support, without any micro-grooves, functionalized by adhesion proteins to control cell orientation. C2C12 cells produce different morphological and cytoskeletal responses to mechanical stimulation depending on their alignment relative to the direction of the cyclic tensile strain: strain applied to 0° micro-pattern lines results in the most irregular actin striation when compared to the highly organized stress fiber orientation observed along the 90° micro-pattern. Myoblast nucleus shape and orientation seem to be determined by geometrical constraints, showing that cyclic tensile strain and geometric constraints may be competing forms of stimuli [225].

#### 3.4.2. Electrospun Scaffolds

##### Effect of Scaffold Structure and Mechanical Properties on Biological Response

The main materials that were used to produce electrospun scaffolds for skeletal muscle engineering are biocompatible and biodegradable synthetic polymers, such as PLGA [186], PCL [189,190,191,196,197,198,260,292], PVDF [187], and polyurethane [184,185,192]. These materials can also be of natural origin such as collagen [188,195,292], gelatin, decorin, silk fibroin, alone or mixed [190,196]. As for the gels, conductive elements can be added to the polymer, such as graphene [195], carbon nanotubes [192,194,198], polyaniline (PANi) [191], or gold nanoparticules [265,275].

Parallel configurations were studied to mimic the natural organization of bundles of aligned muscle fibers, which is necessary to develop high contractile forces [176]. Of the parameters that could be adjusted during the electrospinning process, Li et al. (2007) showed that the rotation speed of the collector had a considerable impact on the anisotropy of the resulting fiber mesh, which in turn, influenced the mechanical properties of the scaffolds [260]. For instance, the tensile moduli for random/aligned fibers of polyurethane (PU) were 2.1 ± 0.4 MPa and 11.6 ± 3.1 MPa, respectively.

It is well-documented that aligned fibers in electrospun scaffolds cause myoblast cytoskeletal reorganization, cell orientation along the fibers, and cell fusion into myotubes, unlike randomly oriented fibers [184,186,187,190]. Physicochemical cues for polymers influence myoblast differentiation, hydrophilic properties, and low matrix stiffness had a beneficial effect on cell response.

Drexler and Powell (2011) investigated coaxial electrospinning methods to produce scaffolds with tunable stiffness and strength without changing the architecture or the surface chemistry. These authors demonstrated that strength and stiffness were positively correlated with the inner core diameter, with no impact on fiber diameter [293]. This method might then make it possible to produce scaffolds with mechanical properties that are similar to those of native skeletal muscle tissue (≈10 kPa) [170]. Furthermore, hybrid composite fibers composed of natural and synthetic polymers are of great interest in order to benefit from the synergistic effect of mechanical properties and the biocompatibility of polymers in the same scaffold [205,294]. Aligned PCL/collagen electrospun fibers, when compared to randomly orientated nanofibers, showed higher tensile strength in scaffolds, as well as effective human myoblast alignment and differentiation into myotubes [265].

The influence of electrospun fiber diameter on skeletal muscle cell behavior remains poorly documented. Liao et al. (2008) produced polyurethane electrospun fibers with various diameters: 600 nm, and 2 µm to 10 µm by varying the polymer concentration (7%, 10%, and 15%). They did not find any influence of electrospun fiber diameter on the differentiation of C2C12 myoblasts [184]. Sreerekha et al. (2013) designed a multiscale composite scaffold with fibrin nanofibers (50–500 nm) and PCL microfibers (1 to 2.5 µm) [295]. These dimensions mimic the hierarchical structure of ECM that is found in native tissues (Figure 2). Topography scale also has an effect on cell responses: hydrogel micro-patterns designed on electrospun materials or wavy imprinted materials improved C2C12 myotube formation, orientation, and length through a multi-dimensional scale [189,197]. A more complex structure has been proposed in the form of a core-shell scaffold that combines aligned nanofiber yarns in a hydrogel shell to provide a suitable 3D environment successfully guiding the C2C12 myoblast alignment and differentiation [196].

Jun et al. (2009) evaluated the effect of PLCL/PANi random fibers on C2C12 myoblast culture. Mechanically, the fibers showed an increase in tensile strength and a decrease in elongation at break as the concentration of PANi increased. While having a minimal effect on the proliferation, the electrically conductive fibers appeared to have a moderate effect on C2C12 cells by increasing the number and length of the myotubes, and enhancing the expression level of myogenic genes [191]. McKeon-Fischer et al. (2011) electrospun PCL with multiwalled carbon nanotubes (MWCNT) and with PAA/PVA hydrogel. The addition of MWCNT increased the mechanical properties of the “actuator” to more than the values of native skeletal muscle. Primary rat muscle cell cultures within a hydrogel were the first to display interactions among actin filaments in the large multinucleated formations [296]. Later, McKeon-Fischer et al. (2014) implanted the same type of scaffold for four weeks on to the *vastus lateralis* muscle of rats. These authors showed that the scaffold displayed early signs of inflammation and fibrotic tissue formation, which decreased over time, while the number of myogenic cells and neovascularization increased, suggesting that this approach could be innovative for muscle repair [297].

##### Effect of Electrical Stimulation on Biological Response

Electrical stimulation was recently investigated on electrospun bioconstructs to simulate motoneuron activity. Ostrovidov et al. (2014) demonstrated the positive effect of administering electric pulses (5 V, 1 Hz, 1 ms) for two days on the maturation and contractility of myotubes from C2C12 cells. These cells were cultured on gelatin electrospun fibers loaded with carbon nanotubes to promote electrical conduction [194]. The same type of results was observed by Sirivisoot and Harrison (2011) on electrospun polyurethane/carbon nanotube scaffolds (5% and 10% *w*/*v* polyurethane), when compared with nonconductive electrospun polyurethane scaffolds after electrical stimulation (Biphasic pulses delivered at 20 Hz) [192].

##### Effect of Mechanical Stimulation on Biological Response

Candiani et al. (2010) used a bioreactor and PU electrospun scaffold to investigate the effect of mechanical conditioning on the development of murine skeletal muscle cells. They applied an unidirectional stretching phase (24 h of stretching at 0.02 mm/h, up to 960 μm of displacement) to mimic bone growth-associated muscle lengthening during embryonic development, followed by a phase of cyclic stretch (frequency 0.5 Hz, amplitude 1 mm). Cyclic stretching induced an eight-fold increase in myosin heavy chain synthesis after 10 days, and contributed to myotube maintenance in a 3D environment [185]. Also, with electrospun PU, Liao et al. (2008) demonstrated that mechanical (5% or 10% cyclic strain at 1 Hz for two days post differentiation) or synchronized electromechanical stimuli (20 V at 1 Hz starting at day 0, 4, or 7 days post differentiation) increased the percentage of striated myotubes from C2C12 cells and an up-regulation of α-actinin and myosin heavy chains. They highlighted the need to carefully consider the combination of topographical and mechanical stimuli to optimize myogenesis. More specifically, these authors showed that a 5% pre-stretching procedure applied after cell seeding and prior to the application of cyclic strain resulted in enhanced myogenic differentiation. They also evidenced that the timing of electrical stimulation application is a crucial factor for modulating myoblast differentiation [184].

## 4. Reconstruction of the Myotendinous Junction

Once a bioengineered tissue has been designed, one of the key challenges for implanting it is its integration into neighboring tissues. Very few studies suggested designing and analyzing biohybrid constructs that mimic the interfaces between two different biological tissues subjected to various mechanical stimuli or strains.

Regarding this aspect, the myotendinous junction (MTJ) is of specific interest. Charvet et al. (2012) reviewed the current understanding of MTJ formation, describing changes during morphogenesis and focusing on the crosstalk between muscle and tendon cells that leads to the development of a functional MTJ. As pointed out, the various mechanisms/events leading to a functional MTJ during embryogenesis are not yet fully understood. However, the structural integrity of MTJs is critical for force transmission from contracting muscle through tendon to bone tissue [298].

The ultrastructure of the MTJ was mostly explored using transmission electron microscopy (TEM), and focused ion beam/scanning electron microscopy (FIB/SEM). At this scale, the MTJ can be described as sarcoplasmic invaginations (ridge-like protrusion), which increase the contact surface between the muscle and tendon. Multidirectional collagen fibers are observed on the tendon side, improving the anchorage between both tissues.

In the past, Larkin’s group [299] attempted to reconstruct the junction while using so-called scaffold-free self-organized tendon constructs (SOT). SOT consisted in collagen-rich deposits and flattened, longitudinally-oriented tenocytes extracted from rat tendons. They were put into contact with pre-established cultures of spontaneously contracting multinucleated myotubes. The interface presented an ultrastructure that resembled the fetal/neonatal MTJ. When subjected to tensile tests, rupture was observed on the muscle side [300]. This approach did not imply a specific scaffold, but it provided new insights into the mechanisms that are responsible for the formation and maturation of the junction, in an attempt to mimic the in vivo conditions.

More recently, Atala’s group proposed two different approaches that are based on a unique scaffold that is composed of three different areas. In a first study, such scaffolds were prepared by electrospinning and consisted in: (i) an area of collagen/PCL fibers, (ii) an interphase area where fibers of collagen/PCL and collagen/PLLA were co-extruded; and, (iii) an area of collagen PLLA fibers. All of the areas were randomly deposited and fiber size was about 500 nm, independently of the electrospun material. Young moduli were around 4, 20, and 28 MPa, respectively. When C2C12 cells were seeded on to PCL, they formed myotubes, while NIH/3T3 fibroblasts spread on PLLA. There was no evidence of cell reorganization at the interface to form a specific MTJ [301]. In a second study, bioprinting was used with thermoplastic PU and C2C12 myoblasts on the muscle side, and PCL and NIH/3T3 fibroblasts on the tendon side. The interface was created by co-localizing the printing of PU and PCL leading to a 10% overlap. After the composite PU–PCL/C2C12-NIH/3T3 construct was printed, the fibrin-based hydrogel bio-ink was cross-linked. The extruded fibers exhibited a diameter of about 300 µm. According to classic tensile tests, the final construct was elastic on the PU-C2C12 muscle side (E = 0.4 MPa), stiff on the PCL-NIH/3T3 tendon side (E = 46 MPa), and intermediate in the interface region (E = 1.0 MPa). Again, both cell lines grew correctly on their respective surfaces and some interfacial features could be observed under confocal microscopy. This type of approach seems quite promising, because it is relatively easy to set up [302]. The next step would be to use more relevant cell types, as well as performing stimulation inducing mechanical stretching to stress the three areas showing the different mechanical properties, thus leading to different mechanotransduction signals.

It can be seen that the literature on the subject is still quite poor, probably because the biological phenomena leading to the formation of the MTJ have not yet been clearly established. Attempts to engineer such junctions could thus also be helpful for fundamental studies in embryology, for instance, to evaluate hypotheses regarding mechanisms that are potentially involved in the development of such a complex structure.

## 5. Conclusions and Perspectives—New Challenges

To conclude, it is obvious that tissue engineering of the musculo-tendinous system is still in its early stages. The investigated protocols summarized in the review are helpful for proposing new perspectives in tendon and muscle healing, which are capable of overcoming the limitations of more classic techniques, such as autologous grafts or more recent purely artificial substitutes or cell therapy. Initially, collagen appeared to be the material of reference, as this fibrillary protein is present both in tendon and muscle. However, the variability of the sources and the various limitations mentioned in this text have led to parallel investigations on synthetic polymers, such as PCL for muscle or PLA, mostly for tendons. Of the shapes used, porous gels and fibers that are produced by electrospinning are the most widely developed. However, there is not yet any consensus regarding the final choice for the material, cell source or stimulation protocol.

Biomimetism or bio-inspiration will probably guide future investigations and this requires in-depth knowledge of the tissue to be reconstructed. In this review, we attempted to follow this process, starting with the biological and mechanical characterization of native tissues (tendon, muscle, and the myotendinous junction), ending with the biological and mechanical outcomes of the reconstructed tissues, as they have been described. Very interestingly, while muscle and tendon might seem quite similar in structure at different scales, they nevertheless present properties that are completely different, as a result of different cell densities (poor in tendon, high with very specialized cells in muscle) and the composition of the ECM.

To date, tissue engineering has designed the scaffold that will host the cells and provide the construct with mechanical properties. In the future, it may be interesting to consider it as a trigger for the “right” cells to produce their own ECM, in a way that is mimicking embryogenesis. Subjected to specific external stimuli, the properties expected of new “smart” materials would thus be different: guiding cell differentiation thanks to their nano/ultrastructure, releasing specific factors on the basis of defined kinetics to mimic the different steps in development, providing signals for cell colonization/differentiation status, or interacting with the new synthesized ECM to provide genuinely hybrid materials with adaptive mechanical properties.

## Figures and Tables

**Table 1:** Material characteristics for tendon tissue engineering.

Material	Scaffold Preparation	Shape and Structure of the Scaffold	Mechanical Properties of the Scaffold	Ref.
Collagen	Freeze drying	Sponges L = 11, 23 or 51 mm 94% porosity, pore size = 62 μm	For L = 23 mm spec. EM = 0.02 MPa Linear Stiffness = 0.05 N/mm Maximum Stress = 0.005 MPa	[38,39]
Collagen/Chondroitin Sulfate		Sponge pore size = 53 μm	Linear Stiffness = 0.025 N/mm	[87]
Collagen/Chondroitin Sulfate		Isotropic sponge pore size = 87 μm Anisotropic pore sizes = 55, 152, 243 μm	ND	[51,55]
Collagen	Extrusion	EDC Crosslinked fiber diameter = 215 µm EDC/EDGE Crosslink diameter = 137 µm	Fiber diameter 215 µm → EM = 19.3 MPa Fiber diameter 137 µm → EM = 46.2 MPa	[52,53]
Collagen	ELAC	Collagen thread diameter = 50–100 μm	ND	[33]
Collagen		Woven collagen scaffold with 81% of porosity	Stiffness = 23.8 N/mm	[34]
PLGA	Electrospinning	Random nanofibers = 568 nm Aligned fibers = 320, 680 and 1800 nm	Random nanofibers → EM = 107 MPa Aligned fibers → EM = 341–510 MPa	[105,106]
PLLA		Aligned nanofiber diameter = 430 nm Random nanofiber diameter = 450 nm	Aligned nanofibers → Stiffness = 3.48 N/mm; EM = 22.76 MPa Random nanofibers → Stiffness = 0.07 N/mm; EM = 0.63 MPa	[30]
PLDLLA		Crimped fiber diameter = 880 nm Amplitude = 5.2 μm Wavelength = 46 μm	Crimped fiber Modulus = 3 MPa	[54]
PEEUR		Aligned or random fiber diameters <1 μm, 1–2 μm or >2 μm	EM = 4.2–9.2 MPa	[45,46]
PCL		Yarned made of twisted aligned fibers (200 μm diameter)	UTS = 17 MPa EM = 30 MPa	[35]
P(LLA-CL)/Collagen		Fiber diameter = 643 nm Final yarn thickness = 150 μm Pore size = 28.5 μm	Yarns EM = 2 MPa Ultimate deformation = 250%	[107]
PLGA	Knitting	Scaffold with 3 yarns. 20 filaments/yarn 25 μm diameter of filament + electrospun nanofibers	Initial failure load = 56.3 N Initial Elastic Stiffness = 5.80 N/mm Initial toe region Stiffness = 0.34 N/mm	[36]
Silk		Combined knitted silk fibers and silk sponge pores size from 20 to 100 μm	Maximum Tensile Load = 252 N Tensile Stiffness = 40 N/mm	[37]
Silk/Collagen		Combined knitted silk scaffold and freeze dryed collagen sponge	Failure force = 21.65 N	[97]

All abbreviations regarding materials can be found in the text. ELAC: Electrochemically aligned collagen fibers.

**Table 2:** In vitro performances of biohybrid scaffold in tendon tissue engineering (↑ = increase, ↓ = decreases).

Cells	Mechanical Stimulation of the Scaffold	Mechanical Properties of Biohybrid Construct	Major Outcomes	Ref.
BMSCs from NZ Rabbit	2 days of static culture and 2.4% strain once every 5 min for 8 h/day for 12 days	Long construct (51 mm): LS = 0.066 N/mm after stimulation. Non-stimulated: LS = 0.047 N/mm	Longest constructs: highest linear stiffness in vitro. Still very weak	[37,38]
BMSCs from NZ Rabbit	2 days of static culture and 2.4% strain 8 h/day for 12 days at 100 or 3000 cycles/day	Stimulated constructs 100 cycles LS = 0.080 N/mm; 3000 cycles LS = 0.032 N/mm	100 cycles/day: ↑ linear stiffness 3000 cycles/day: ↑ mRNA levels of *Col1* and *Col3*. ECM not shown	[87]
Primary horse tenocytes	None	ND	Anisotropic sponges: ↑ cell number, alignment and metabolic activity Pores >150 μm: ↑ cell proliferation and activity Smaller pores with high crosslinking density: ↑ differentiation	[51,55]
Sheep patellar tendon fibroblasts	None	ND	EDC/EDGE crosslinking: better mechanical properties, proliferation but ↓ cell viability EDC cross-linked fibers ↑ ECM production	[52]
Human MSCs	None	ND	ELAC threads: ↑ cell adhesion, ↓proliferation, ↑ tendon differentiation compared to random threads	[32]
Human BMSCs	None	ND	Cells aligned in the 3D structure. Up-regulation of tendon-related markers (TNMD and COL1). New matrix deposition	[33]
Human Rotator Cuff Fibroblasts	None	For 600 nm diameter, after 14 days: Aligned Constr: EM = 341 MPa, Random Constructs: EM = 107 MPa	Aligned/random scaffolds: No differences in cell proliferation or cell matrix deposition Nanofiber: ↑ cell proliferation and matrix synthesis Microfiber: ↑ tendon-like gene markers	[105,106]
Human TSPC from foetal Achilles Tendon	None	ND	Aligned scaffolds: ↑ tendon differentiation (aligned cells and expression of COL1, SCX, Eya2)	[29]
Bovine fibroblasts	Short term: 10% of cyclic uniaxial strain at 1 Hz 3 h/day. Long term: 3 h/day at 1 Hz in alternate days for 2/4 weeks	After 4 weeks on dynamic culture: Crimped structures EM = 33 MPa Uncrimped structures EM = 17 MPa For non-stimulated culture: uncrimped EM = 8.7 MPa	Crimped-like fibers: ↑ collagen accumulation Dynamic culture: ↑ ECM production (collagen and proteoglycans)	[54]
C3H10T1/2	2 days static culture + 3 days static (50 mN)/dynamic load (4% strain 0.25 Hz for 30 min)	ND	Static load, larger fibers, non-alignment: ↑ tenogenic differentiation	[45,46]
Human BMSCs	5 days of static culture. Cyclic uniaxial strain at 5% elongation at 1 Hz 1 h/day for 7 or 21 days	After 21 days on dynamic culture, UTS = 50 MPa; EM = 110 MPa. Under dynamic culture UTS = 20 MPa; EM = 110 MPa	aligned fibers: ↑ cell alignment Uniaxial cyclic strain: ↑ tendon-related markers (COL1, COL3, TNC, FN)/unloaded cells	[34]
Rabbit tendon cells	Static culture for 1 day. Cyclic uniaxial strain at 4% elongation at 0.5 Hz 2 h/day for 14 days	ND	Dynamic culture: ↑ Tendon related markers (COL1, COL3, decorin, TNC, Biglycan and ↓ of bone (Runx2) or cartilage related markers (COL2). Cells aligned in both static or dynamic culture	[107]
Pig BMSCs	None	Failure load = 1. 82 N; Elastic Stiffness = 0.64 N/mm; Toe Region Stiffness = 0.05 N/mm	knitted structure + electrospun nanofibers: ↑ cell proliferation, collagen production and tendon-related markers (COL1, Decorin, Biglycan)	[35]
Human BMSCs	None	Tensile Load = 257 M Tensile Stiffness = 50 N/mm	Combined silk scaffolds with cells shows higher proliferation, ECM production (COL1, COL3 and GAGs) than knitted silk scaffolds.	[36]
Rabbit TSPCs	None	ND	No difference in cells attachment, spreading and proliferation Aligned collagen sponges → aligned ECM deposit	[97]

**Table 3:** In vivo performances of biohybrid construct in tendon tissue engineering (↑ = increase).

Animal Model, Tissue Site and Duration of Implantation	Mechanical Stimulation before Implantation	Mechanical Properties of the Biohybrid Construct Following Implantation	Biological Outcomes	Ref.
Rabbit patellar tendon 12 weeks	2.4% strain every 5 min for 8 h/day for 12 days prior implantation	Stimulated repair: LS = 241.6 N/mm; EM = 441.2 MPa. Non-stimulated repair: LS = 88.6 N/mm; EM = 343.2 MPa	Stimulated repair constructs: ↑ mechanical properties over time than non-stimulated repair	[38]
Sheep patellar tendon 3 or 6 months	None	After 6 months: EDC cross linked: EM = 73 MPa EDC/EDGE cross linked: EM = 68 MPa	EDC cross-linked fibers: ↑ mechanical properties, integration, resorption and tissue ingrowth after 6 months	[53]
-Mice muscle for 1 or 6 weeks -Mice skin for 1 week	None	None	-Cytotoxicity model: aligned cells with more oriented bundles of collagen compared to random scaffolds -Subcutaneous model: ↑ concentration of collagen with aligned morphology in aligned scaffolds	[30]
-In vivo: Mice back for 2, 4 or 8 weeks -In situ: Rabbit tendon for 4 or 12 weeks	In situ: Static or dynamic culture, 4% elongation at 0.5 Hz 2 h/day, 14 days	In situ: EM = 426.69 MPa for dynamic group EM = 41.5 MPa for static group	-In vivo: Mechanical stimulation: ↑ neo-tendon tissue formation with aligned ECM deposition -In situ: Dynamic culture: ↑ alignment of cells and matrix deposition. Larger collagen fibers on pre-stimulated construct	[107]
Rabbit tendon 12 weeks	None	Failure force = 139.85 N Stress at failure = 4.34 MPa Energy = 0.42 J Stiffness = 26.67 N/mm	Combined knitted and collagen-aligned sponge: ↑ ovoid cells, larger and denser collagen fibers	[97]

**Table 4:** Materials characteristic for muscle tissue engineering.

Material	Scaffold Preparation	Shape and Structure of the Scaffold	Mechanical Properties of the Scaffold	Ref.
Collagen I	Hydrogel (Layer)	Membrane Flexcell	EM = 930 kPa	[174]
Collagen		Sheet -smooth	ND	[173]
Collagen I—Matrigel		Layer	ND	[200]
Fibrin		Layer	ND	[175]
Collagen I		3D cylinder hydrogel with inner diameters: 0.90 and 0.53 mm	ND	[176]
Oxidized alginate/gelatin cross-linking		Layer	EM = 1 and 10 kPa	[177]
PMDS/NCO-sP(EO-*stat*-PO) hydrogel/fibronectin coating		Fibronectin lines micropattern (30 μm wide parallel lines with 40 μm spacing) coating on hydrogel	EM ~1 MPa	[225]
PDMS/laminin coating		Micropatterned waves with 3, 6 and 12 μm in periodicity	ND	[178]
PDMS/fibronectin coating		Fibronectin geometrical cues: linear, 30°, circular micropatterns	EM = 100 and 500 Pa	[220]
poly-l-lactide/trimethylene carbonate		Micropatterns with groove widths (5, 10, 25, 50, 100 μm) and depths (0.5, 1, 2.5, 5 μm)	ND	[180]
Gelatin methacryloyl	Hydrogel (3D matrix)	Hydrogel slabs cross sections: 2000 μm × 2000 μm, 1000 μm × 1000 μm, 500 μm × 500 μm	Compressive modulus = 1 to 17 kPa	[182]
Gelatin methacrylate		Micropatterns with groove-ridges: 100 μm/50 μm; 100 μm/100 μm	ND	[183]
Mix of matrigel and fibrin		3D matrix: 1.5 mm thick—hexagonal holes lengths = 0.6, 1.2, or 1.8 mm	ND	[204]
Mix of collagen and matrigel		3D matrix	ND	[203]
Fibrin		None	ND	[202]
ECM proteins		3D matrix	EM = from 200 to 500 kPa; Passive tension = from 860 to 1150 μN	[286]
Polycarbonate polymer and titanium with gold nanoparticulates	Hydrogel (3D porous sponge)	Micropatterns with ridges, grooves, arrays of holes (5–75 μm)	ND	[181]
l-lactide/e-caprolactone copolymer (70/30)		Porous sponge = 3 cm diameter, 2–3 mm thickness with an average pore size of about 320 μm	ND	[201]
Atelocollagen		Porous sponge = pore diameters with a range of 50 to 100 μm	ND	[243]
Collagen		Porous sponge	ND	[199]
Polyurethane	Electrospinning	Smooth film or random or aligned fibers Aligned fiber size = 600 nm–10 µm	EM = 0.5–1–22 MPa	[184]
Polyesterurethane (DegraPol^®^)		Highly oriented fiber (10 µm diameter) Scaffold thickness = 200 µm	ND	[185]
PCL		Highly oriented fibers = 438–520 nm range	Non-aligned scaffolds = EM 2.1 MPa	[260]
PLGA		1500 rpm: 0.6–0.9 μm range oriented with standard deviation: 19.5° 300 rpm: 0.4–0.8 μm range random with standard deviation: 74.7°	ND	[186]
ß-PVDF		Fiber diameter = ~200 nm Films with a thickness = ~110 µm	ND	[187]
Collagen I		Spring-shape	ND	[188]
Chitosan/PVA		Random structure: diameter = 137 nm, pore size = 1.9 µm^2^	Break strain = 83.42%, Peak stress = 6.63 MPa	[205]
PCL		Parallel -oriented with wavy micropatterns: period. = 90um—depth = 14um—fiber diam. = 148 nm random orientation: size fibers = 265 nm aligned fibers: size fibers = 354 nm	EM = 36 MPA; UTS = 15 MPa; Elongation to break = 44% EM = 7 MPa; UTS = 4 MPa; Elongation to break = 161% EM =17 mMPa; UTS = 14 MPa; Elongation to break = 64%	[189]
PCL blends with PLGA or decorin		Aligned fiber diameters from 0.4–0.7 µm to 0.7–2.7 µm, for 15% *w/v* and 20% *w/v* of polymer solution	ND	[190]
PCL/PANi: (100/0); (85/15); (70/30)		Random 3D interconnected pores or oriented fibers Fiber diameters: PLCL/PANi (100/0) = 516 nm PLCL/PANi (85/15) = 499 nm PLCL/PANi (70/30) = 466 nm	Tensile strain—Elongation at break—EM—conductivity: PLCL/PANi (100/0): 18.2 MPa—248%—4.74 MPa PLCL/PANi (85/15): 16.7 MPa—176%—6.8 MPa—0.160 ± 0.046 S/cm PLCL/PANi (70/30): 14.1 MPa—160%—6.41 MPa—0.296 S/cm	[191]
Polyurethane/carbon nanotubes		Thickness = 36–64 µm range; Fiber diameter = 441–1533 nm range; Pore area = 2.5–12.3 µm^2^	EM = 6.1–41.0 MPa range Tensile strength = 9.95–45.02 MPa range; Elongation at break = 115–300% range	[192]
Gelatin crosslinked by GTA, +/−0.5 or 5 mg/mL MWNTs		Fiber diameter from 18 kV = 250 to 900 nm and from 15 kV = 300 to 600 nm	EM (20% Gelatin) = 509 ± 37 kPa EM (20% gelatin −0.5 mg/mL MWNTs) =1170 kPa EM (20% gelatin −5 mg/mL MWNTs) = 1170 kPa	[194]
PLGA/collagen with graphene oxide nanoparticules		Randomly oriented average diameter = 440 nm	Hydrophilicity angle contact = 85°; Surface energy = 32.35 mN/m; Tensile strenghs = 16.8 MPa; E = 460 MPa	[195]
PCL/collagen sputter-coated with gold nanoparticules		Fiber diameters = from 296 to 334 nm Fiber orientation: - Random parallel - Random perpendicular - Aligned parallel - Aligned perpendicular	Tensile strength—Elongation at break EM: Random parallel: 4.01 MPa—53%—4.33 MPa Random perpendicular: 3.86 MPa—53%—4.07 MPa Aligned parallel: 4.88 MPa—42.33%—4.43 MPa Aligned perpendicular: 3.06 MPa—91.67%—42.93 MPa	[265]
Fibers:PCL/silk fibroin/polyaniline Hydrogel: PEG		Aligned fiber diameters within hydrogel = 600 to 900 nm Yarn diameters within hydrogel = 50, 100, 165 µm	Tensile stress = 1.49 to 4.02 cN by yarn diameter: 25 to 165 µm Strain of yarns with diameters from 76% to 107%,	[196]
PCL/multiwalled carbon nanotubes (MWCNT) Hydrogel: PAA/PVA		Fiber diameter averages: PCL: 1.032 µm PCL-MWCNT: 1.704 µm PCL-MWCNT-Hydrogel:1.861 µm	Electrical conductivity PCL: 0.026 S/cm PCL-MWCNT:0.043 S/cm PCL-MWCNT-Hydrogel: 0.039.011 S/cm	[296]
PCL Hydrogel: PEG		Random, parallel, perpendicular fibers versus hydrogel pattern; Hydrogel pattern: 100 and 200 μm width	ND	[197]

**Table 5:** In vitro performances of biohybrid construct in muscle tissue engineering (↑ = increase, ↓= decrease).

Cells	Mechanical and/or Electrical Stimulation	Biological Outcomes	Ref.
C2C12	Mechanical: uniaxial cyclic tensile strain (CTS)—semi-sinusoidal tensile stretching pulses with a duration of 1 s. Peak amplitude 15%	Cell alignment perpendicular to the direction of strain ↑ myotube/myoblast ratio and % of myosin-positive myotubes	[174]
C2C12	Mechanical: 24 h of static culture Electrical: period 1 s, duration 0.1 s for 72 h, amplitude: 0.1 V to 12 V	Pulses lower than 8 V: ↑ cell adherence and proliferation Pulses of 0.1 V: ↑ cell differentiation Cell repetitive contraction at 8 days	[173]
MPCs/C2C12	Electrical: 4 V/cm, 6 ms pulses, frequency 2 Hz for 48 h	↑ sarcomere assembly and expression of late muscle maturation markers Faster maturation of myotubes in 3D model system than in 2D MPCs more mature than C2C12 and more susceptible to the electrical stimulation	[200]
MPCs/C2C12	Mechanical: 2 days uniaxial ramp stretch of 0–2% followed by an uniaxial intermittent stretch regime of 2–6% (3 h on, 3 h off)	↓ maturation into functional muscle fibers	[175]
C2C12	Mechanical: Cyclic stretching of 60 Hz −5% amplitude for 4 days	↑ degree of cell orientation and differentiation. Formation of a necrotic core in larger diameter rode	[176]
MSCs	-	Coverage of the total surface hydrogels OA/GEL (30/70) after 14 day culture	[177]
C2C12	Mechanical: orientation relative to the cyclic strain direction: 0°–45°–90°, amplitude 7% at 0.5 Hz for 4 days	Alignment of the actin stress fibers relative to the strain direction Significant effect on stress fiber orientation under geometric constraints of 30 μm width	[225]
C2C12	-	Wave periodicity (6 µm) of scaffold: ↑ alignment of moyblasts and myotubes	[178]
C2C12	Electrical: 20 V, 50 ms pulse, 1 Hz	30° hybrid structure: ↑ differentiation into myotubes with the highest fusion index	[220]
C2C12	-	↑ cell differentiation and maturation with 25 μm grooves width and 0.5–1 μm depth after 7 days of culture	[180]
C2C12	-	GelMA 3 and 4%: ↑ myogenesis Hydrogel structures (500 μm × 500 μm) and (1000 μm × 1000 μm) ↑ cell parallel orientation	[182]
C2C12	Electrical: 48 h of stimulation at 22 mA,1 Hz, and 2 ms	Surface topography with ridge width 50 µm: ↑ myotube orientation compared to width of 100 µm Electrical stimulation ↑ myoblast alignment and myotube diameter	[183]
Neonatal rat skeletal myoblasts	-	Elongated pores: ↑ cell alignment Tissue networks: ↑ fraction of myogenin-positive nuclei, and cell maturation into myotubes	[204]
Primary human skeletal cells	Mechanical: 3 sets (5% strain for 2 days,10% strain for 2 days and 15% strain for 4 days) of 5 stretch/relaxation cycles, each separated by 30 s of rest, with 28 min of rest after the third set	Repetitive stretch/relaxation cycles: ↑ myofiber diameter, area percentage and aligned multinucleated myofibers	[203]
Primary rat myoblast	Electrical: biphasic stimulation 6.8 mA; 4 ms. Electric bursts lasted for 250 ms, delivered at intervals every 4 s	↓ expression of the MRFs, MyoD and myogenin and AChR-ε	[202]
C2C12	Electrical: bipolar pulses: 20 V, amplitude (21.6-V cm^−1^ field strength) and 50 ms pulse	IGF-1: ↑ rate of fusion, maturation and myotube density Electrical stimulation triggered contraction	[286]
C2C12/primary myoblast	-	Microscale topography: modulates myoblast alignment	[181]
Human myoblast	-	↑ desmin and MyoD expression and myotube formation	[201]
MPCs	Electrical: Pulses 70 mV/cm for 3 ms, frequency 33.3 mHz	↑ expression of MyoD and desmin compare to non-stimulated control and ↑ total amount and release rate of NO_X_	[199]
C2C12	Electrical: 20 V, 1 Hz, for 1 h with 5 h of rest Synchronized electromechanical: pre-stretching mechanical protocol: 5% cyclic strain at 1 Hz, followed by electrical stimulation	↑ degree of myotube striation when applied during post differentiation period compared to prior one Synchronized elecromechanical stimulation ↑ degree of myotube striation compared to unstimulated control	[184]
C2C12	Mechanical: 5 days of static culture (24 h of stretching at 0.02 mm/h, up to 960 μm displacement) followed by stretching pattern (frequency 0.5 Hz, amplitude 1 mm, 30 sec rest, followed by 28 min rest)	Cyclic stretching pattern stimulation: ↑ myosin accumulation	[185]
C2C12	-	Parallel electrospun fibers ↑ myoblast alignment, myosin expression and sarcomeric protein organization	[186]
C2C12	-	Negative poled ß-PVDF ↑ cell adhesion and proliferation. Oriented ß-PVDF fibers ↑ cell alignment	[187]
C2C12	-	Stained MHC-positive cells at day 7, multi-nucleated with parallel orientation along the microfiber at day 10 Myoblasts showed typical sarcomeric cross-striations The entire tissue continuously pulsated by autonomous contraction	[188]
Rabbit MSCs	-	Hybrid (chitosan/PVA) composition: ↑ myogenesis	[205]
C2C12	-	Periodic grooves: ↑ myotube formation and orientation	[189]
C2C12	-	Aligned PCL/PLGA 50% fibers: ↑ cell growth and differentiation versus to randomly oriented fibers Decorin addition: ↑ cell fusion, myotube length but ↓ myotube alignment	[190]
C2C12	-	PLCL/PANi (85/15) and (70/30): ↑ myotube length and width and ↑ expression of *myogenin*, *troponin T* and *MHC* genes	[191]
C2C12	Electrical: 10 µA at 10 Hz, 6 h/day, 21 days	Modulation of myotube maturation depend on the conductivity of the scaffolds	[192]
C2C12	Electrical: 5 V, 1 Hz, 1 ms for 2 days	↑ speed and the rate of myotube formation and length ↑ myogenin and FAK gene expression Increasing carbon nanotube concentration ↑ maturation and contractibility of myotubes	[194]
C2C12	-	GO-PLGA-Col hybrid scaffold composition ↑ cell attachment and proliferation, myogenic differentiation, myoblast fusion and myotube maturation	[195]
C2C12	-	Hybrid scaffold/hydrogel: ↑ formation of 3D aligned and elongated myotube ↑ Cell adherence, alignment and elongation with 50 and 100 µm yarns embedded in hydrogels	[196]
C2C12	-	PCL-carbon nanotubes-hydrogel: ↑ multinucleated cellular formation	[296]
C2C12	-	Aligned nonofibers: ↑ cells elongation compared to random and perpendicular nanofibers 100 μm pattern sizes on parallel fibrous scaffolds ↑ MHC expression and myogenesis	[197]

## Abbreviations

EDC	(1-ethyl-3-(3-dimethylaminopropyl) carbodiimide hydrochloride
ASC	Adipose stem cell
ATMP	Advanced therapy medicinal products
bFGF	Basic fibroblast growth factor
BMSC	Bone marrow stem cell
BMP	Bone morphogenetic protein
JNK	c-Jun NH2-terminal kinases
camk	calmodulin-dependent protein kinases
calc	Calcineurin
Calp	Calpain
COL	Collagen
CTGF	Connective tissue growth factor
CTS	Cyclic tensile strain
DF	Dermal fibroblast
EM	Elastic modulus
ELAC	Electrochemically-aligned collagen
EDGE	Ethylene-glycol-diglycidyl-ether
ECM	Extracellular matrix
ERK	Extracellular signal-regulated kinases
Eya	Eye absent homolog
FAP	Fibro-adipogenic progenitors
FGF	Fibroblast growth factor
FAK	Focal adhesion Kinase
FIB	Focused ion beam
GelMA	Gelatin methacryloyl
GPa	GigaPascal
T*g*	Glass transition
GO	Graphene oxydative
GDF	Growth and differentiation factor
HGH	Hepatocyte growth factor
IGF	Insulin-like growth factor
IL	Interleukin
LS	Linear stiffness
MKKs	McKusick-Kaufman syndrome
MSC	Mesenchymal stem cell
p38	Mitogen-activated protein kinases
MWCNT	Multiwalled carbon nanotubes
MPCs	Muscle progenitor cells
MRF	Myogenic regulatory factor
MHC	Myosin heavy chain
MTJ	Myotendinous junction
NHS	*N*-Hydroxysuccinimide
OA	Oxidized alginate
PI3K	Phosphatidylinositol-3-kinase
PDGF	Platelet derived growth factor
PEEUR	Poly (esterurethane urea)
PCL	Poly (ε-caprolactone)
PEG	Poly ethylene glycol
PLGA	poly lactic-co-glycolic acid
PLLA	Poly-l-lactic acid
PAA	Poly(acrylic acid)
PCL	Poly(caprolactone)
	Poly(l-lactide-co-d,l-lactide)
PLA	Poly(l/d)lactide
PVA	Poly(vinyl alcohol)
PLGA	Poly[(lactic acid)-co-(glycolic acid)]
PANi	Polyaniline
PDMS	Polydimethylsiloxane
PGA	Poly(glycolic acid)
PU	Polyurethane
PVDF	Polyvinylidene fluoride
PG	Proteoglycan
Scs	Satellite cells
SEM	Scanning electron microscopy
SCX	Scleraxis
SDS	Sodium dodecyl sulfate
sGC	Soluble guanylyl cyclase
TNC	Tenascin-C
TDSC	Tendon derived stem cell
TSPC	Tendon stem/progenitor cells
TNMD	Tenomodulin
TE	Tissue engineering
TGF-β	Transforming growth factor-β
TEM	Transmission electron microscopy
TnBP	Tri(n-butyl)phosphate
UTS	Ultimate tensile strength
VEGF	Vascular endothelial growth factor
VMLs	Volumetric muscle losses
YM	Young modulus (E)
